# Self-assembled nanoparticles from Xiexin Decoction attenuate ulcerative colitis by targeting VDAC1-Mediated NLRP3 inflammasome activation

**DOI:** 10.1016/j.mtbio.2026.103078

**Published:** 2026-03-27

**Authors:** Zheng Chu, Tong Yang, Ying Zhang, Qianru Zhu, Dawei Wang, Hechen Tang, Guoxin Zhang, Mengyao Jiang, Lirun Zhou, Liting Xu, Tianyun Fan, Junzhe Zhang, Ang Ma, Chengchao Xu, Jigang Wang, Huan Tang

**Affiliations:** a State Key Laboratory for Quality Ensurance and Sustainable Use of Dao-di Herbs, Artemisinin Research Center, and Institute of Chinese Materia Medica, China Academy of Chinese Medical Sciences, Beijing, 100700, China; b Department of Pulmonary and Critical Care Medicine, Shenzhen Institute of Respiratory Diseases, Guangdong Provincial Clinical Research Center for Geriatrics, Shenzhen Clinical Research Center for Geriatrics, Shenzhen People's Hospital, The Second Clinical Medical College, Jinan University, Shenzhen, Guangdong, 518020, China; c Institute of Nanotechnology And Intelligence (inAI), College of Chemistry and Material Sciences, Jinan University, Guangzhou, 511443, China

**Keywords:** Ulcerative colitis, Self-assembled nanoparticles, Berberine, VDAC1, Anti-inflammation, Activity-based protein profiling

## Abstract

Ulcerative colitis (UC) remains a clinical challenge due to limited efficacy and side effects of conventional therapies, highlighting the need for novel interventions. Here, we investigate the therapeutic potential of naturally derived self-assembled nanoparticles (XDNPs) from the traditional Chinese medicine formula Xiexin Decoction (XXD) and rationally designed carrier-free nanoparticles (RBNPs) composed of berberine (BBR) and rhein. Both formulations effectively mitigated DSS-induced colitis by normalizing gut dysbiosis, reducing the release of pro-inflammatory mediators and facilitating macrophage phenotypic switching. Mechanistically, activity-based protein profiling identified VDAC1 as a direct functional target of BBR. Binding to VDAC1 inhibits its oligomerization, preventing the cytosolic release of oxidized mitochondrial DNA and subsequent activation of the NLRP3 inflammasome in macrophages. Co-assembly with rhein further enhances cellular uptake and bioavailability, amplifying anti-inflammatory efficacy. Proteomic and molecular analyses confirmed broad modulation of immune and metabolic pathways, while *in vivo* safety assessments demonstrated excellent biocompatibility without detectable organ toxicity. Collectively, this work elucidates a VDAC1-mediated mechanistic axis underlying the potent therapeutic effects of BBR-containing nanoparticles and establishes a rational nanomedicine strategy inspired by traditional herbal formulations. These findings provide a promising platform for the development of targeted, biocompatible nanotherapeutics for UC and other inflammatory disorders.

## 1 Introduction

Ulcerative colitis (UC) is a chronic inflammatory bowel disease (IBD) characterized by recurrent intestinal inflammation, disruption of the epithelial barrier, and dysbiosis of the gut microbiota [1]. Globally, both the incidence and prevalence of UC are increasing, posing a growing challenge to public health systems [1,2]. The pathogenesis of UC involves a complex interplay among genetic susceptibility, environmental triggers, immune dysregulation, and alterations in the gut microbiome [3]. This multifactorial etiology complicates clinical management and contributes to frequent disease relapse and an elevated risk of colorectal cancer [4,5]. Current standard therapies, as recommended by the American Gastroenterological Association, include 5-aminosalicylic acid (5-ASA), corticosteroids, immunomodulators, and biologic agents such as *anti*-TNF-α antibodies. However, these treatments often yield suboptimal outcomes, with fewer than 60% of patients achieving sustained clinical remission [6,7]. Moreover, long-term use of these agents is associated with significant adverse effects and progressive loss of therapeutic efficacy [8,9]. Therefore, there is an urgent need for novel, multitargeted therapeutic strategies that provide improved efficacy and safety in UC management.

Traditional Chinese Medicine (TCM) offers a holistic, multi-component, and multi-target therapeutic paradigm that has attracted growing scientific interest for treating complex, multifactorial diseases such as UC [10,11]. Unlike single-target drugs, TCM formulations can concurrently modulate multiple pathological pathways, thereby addressing the limitations of conventional therapies. In the context of UC, accumulating clinical and preclinical evidence indicates that TCM interventions can alleviate intestinal inflammation, slow disease progression, reduce relapse frequency, and enhance patients' quality of life [12,13]. One representative example is Xiexin Decoction (XXD), a classical herbal formula first recorded in the ancient medical text Treatise on Febrile Diseases. Comprising *Rheum palmatum* L., *Coptis chinensis* Franch., and *Scutellaria baicalensis* Georgi, XXD has been officially included in China's “List of Ancient Classical Prescriptions” and has a long history of use in treating gastrointestinal disorders [14]. According to TCM theory, XXD functions to “clear heat, dry dampness, and relieve pain”, corresponding well with the pathological manifestations of active UC [[14], [15], [16]]. Modern pharmacological studies have begun to elucidate the scientific basis for these traditional principles. The therapeutic effects of XXD are largely attributed to the synergistic actions of its bioactive constituents. For instance, berberine, a major isoquinoline alkaloid in *Coptis chinensis*, has been shown to attenuate experimental colitis and preserve intestinal epithelial integrity by modulating key signaling pathways such as NF-κB and the NLRP3 inflammasome [[17], [18], [19]]. Similarly, rhein, a predominant anthraquinone derived from *Rheum palmatum*, mitigates intestinal hyperpermeability, regulates gut microbiota composition, and suppresses local inflammation [[20], [21], [22]]. Collectively, these findings suggest that XXD alleviates UC through integrated mechanisms involving immunomodulation, microbiota restoration, and mucosal repair, thereby exemplifying the multi-target therapeutic potential of TCM.

Despite their well-documented therapeutic potential, a major challenge in the pharmacological study of traditional herbal decoctions such as XXD lies in the precise enrichment of active constituents and, more fundamentally, the unequivocal identification of their therapeutic material basis [23,24]. Recent advances at the interface of nanoscience and TCM research are transforming this understanding. Increasing evidence indicates that herbal decoctions are not merely simple aqueous solutions of small molecules, but rather complex, multiphase dispersion systems that contain abundant, naturally occurring, self-assembled nanoscale supramolecular aggregates [[25], [26], [27]]. These nanostructures are thought to form spontaneously during the decoction process through molecular self-assembly, driven by intermolecular interactions among diverse phytochemicals [28,29]. Importantly, these supramolecular entities can act as intrinsic nanocarriers, markedly enhancing the solubility and stability of poorly water-soluble compounds, such as lipophilic molecules [30]. This intrinsic nanoarchitecture aligns with the established paradigm of nanomedicine, which has proven to be a promising strategy for managing inflammatory diseases [[31], [32], [33]]. Emerging evidence further suggests that these herbal-derived nanostructures can facilitate gastrointestinal absorption, thereby improving the overall bioavailability and pharmacological efficacy of the contained phytochemicals [34,35]. This paradigm shift, recognizing the intrinsic nanoarchitecture of herbal decoctions, provides a novel conceptual framework for redefining the material basis of TCM and elucidating the complex, multitarget mechanisms that have historically resisted deconvolution using conventional pharmacological approaches [25,26,36].

**Scheme 1:**
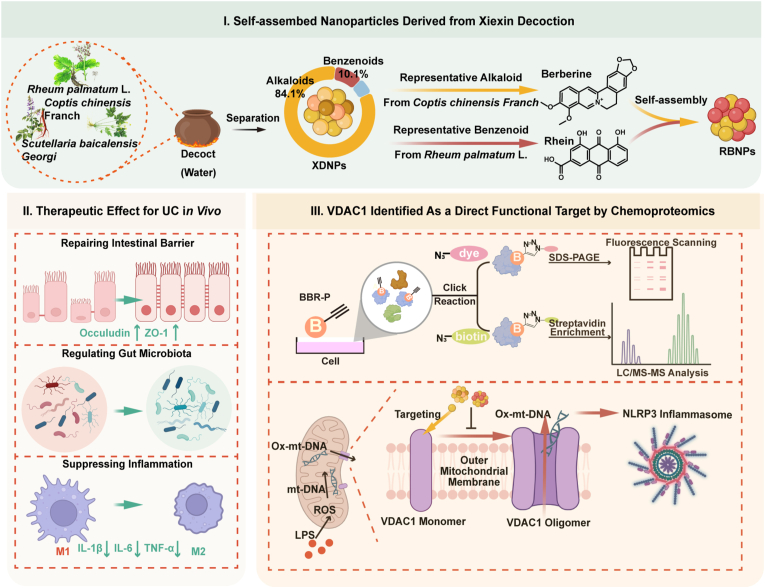
Nano-TCM strategy for investigating the protective mechanisms of self-assembled herbal nanoparticles derived from Xiexin Decoction (XXD). Naturally occurring XDNPs were identified as the primary active constituents of Xiexin Decoction (XXD) against ulcerative colitis. Both XDNPs and the derived binary nanoparticles (RBNPs), self-assembled from BBR and rhein, demonstrated superior efficacy in alleviating colitis symptoms, restoring intestinal barrier integrity, and rebalancing gut microbiota compared with the conventional decoction. Mechanistically, BBR within the nanoparticles directly targets VDAC1 on the mitochondrial outer membrane, inhibiting its oligomerization and preventing the cytosolic release of oxidized mtDNA. This blockade suppresses NLRP3 inflammasome activation, reduces pro-inflammatory cytokine production, and mitigates colonic inflammation, establishing a direct molecular basis for the therapeutic effects of XXD-derived nanoparticles.

Building on the emerging recognition of self-assembled nanostructures in herbal decoctions, the present study aimed to systematically isolate and characterize the naturally occurring nanoparticles within XXD (termed XDNPs) and evaluate their therapeutic potential against UC. We hypothesized that these endogenous self-assemble nanostructure constitute a key pharmacologically active fraction of the decoction. To test this hypothesis, we employed a dextran sulfate sodium (DSS)-induced murine colitis model to compare the therapeutic efficacy of XDNPs with that of the crude XXD extract. Furthermore, to elucidate the underlying molecular mechanisms and identify direct protein targets, we integrated proteomics analyses with activity-based protein profiling (ABPP) in macrophages, a central immune cell type in UC pathogenesis. Our findings revealed several critical insights (Scheme 1). First, we successfully isolated XDNPs from the aqueous XXD extract and demonstrated their superior efficacy in ameliorating colitis symptoms, restoring intestinal barrier integrity, and rebalancing gut microbiota compared with the conventional decoction. Second, inspired by the dominant chemical composition of XDNPs, we designed and synthesized binary nanoparticles (RBNPs) via self-assembly of berberine (BBR) and rhein, the representative alkaloid and benzenoid constituents, respectively. These synthetic RBNPs not only replicated but, in some aspects, surpassed the therapeutic benefits of natural XDNPs, thereby validating the functional contribution of these key components. Most notably, our mechanistic investigations uncovered a previously unrecognized signaling axis through which these nanoparticles exert their anti-inflammatory effects. Using ABPP approach, we identified the voltage-dependent anion channel 1 (VDAC1) located on the mitochondrial membrane as a direct molecular target of BBR. We further demonstrated that BBR binding inhibits VDAC1 stress-induced oligomerization, preventing the release of mitochondrial DNA (mtDNA) into the cytosol. This inhibition consequently suppresses activation of the NLRP3 inflammasome, a central driver of UC-related inflammation. Collectively, this work not only identifies a novel nano-active component within a classical TCM formula but also elucidates its precise molecular mechanism of action, establishing a scientific foundation for the modernization of herbal medicine through nanotechnological perspectives.

## 2 Materials and methods

### 2.1 Materials

Vital River Laboratory Animal Technology Co., Ltd. (Beijing, China) supplied the male C57BL/6 mice (aged 6–8 weeks). Flow cytometry antibodies were procured exclusively from BioLegend. The Cell Counting Kit-8 (CCK-8) was sourced from the Tongren Institute of Chemical Research. Sigma-Aldrich supplied Lipopolysaccharide (LPS), and other biochemicals including dithiothreitol (DTT), iodoacetamide (IAA), and 2′,7′-dichlorodihydrofluorescein diacetate (DCFH-DA). We obtained therapeutic compounds such as 5-Aminosalicylic acid (5-ASA), rhein, and BBR from Aladdin Scientific, while MP Biomedicals provided Dextran sulfate sodium (DSS, Mw: 36,000–50,000 Da). MedChemExpress supplied the Cy5.5 dye. Cloud-Clone Corp provided recombinant human VDAC1. ELISA kits targeting TNF-α, IL-1β, and IL-6 were acquired from Shanghai Enzyme-linked Biotechnology Co., Ltd. Thermo Fisher Scientific was the source for NeutrAvidin Agarose Resin, and TMT 10plex Mass Tag reagents. For molecular analysis, we utilized the FastPure Cell DNA Isolation Mini Kit (DC102-01) from Vazyme and the mitochondria isolation reagent (C3601) from Beyotime. TransGen Biotech supplied the TRIzol reagent, Top Green qPCR SuperMix, and the One-Step gDNA Removal and cDNA Synthesis SuperMix (AT311). Simulated gastric fluid (SGF, pH 1.2) and simulated intestinal fluid (SIF, pH 6.8) were purchased from Shanghai Yuanye Bio-Technology Co., Ltd.

### 2.2 Preparation of XDNPs

A traditional water immersion method was employed to prepare the herbal decoction from a mixture of *Rheum palmatum* L., *Coptis chinensis* Franch., and *Scutellaria baicalensis* Georgi in a 2:1:1 ratio. The mixture was boiled, and the decoction was filtered through gauze to remove residues, followed by low-speed centrifugation (1000 rpm). The resulting supernatant was freeze-dried to obtain XXD powder. To isolate the nanoparticle fraction, the supernatant was centrifuged at high speed (4000 rpm), and the precipitate was collected. The nanoparticles were resuspended in double-distilled water, dialyzed against water for 12 h using a dialysis membrane (molecular weight cutoff, MWCO: 3.5 kDa), and then freeze-dried to yield XDNPs.

### 2.3 Preparation of RBNPs

We fabricated Berberine-rhein nanoparticles (RBNPs) utilizing a self-assembly protocol wherein 10 mM methanolic BBR and 10 mM rhein in DMSO were mixed at a 1:1 molar equivalent. The solution's acidity was adjusted to pH 7.0–7.5 by adding 1 M sodium hydroxide under vigorous stirring conditions for 0.5 h. The resulting pre-mixture was slowly infused into warm PBS (60 °C) with continuous agitation, followed by 30 min of bath sonication. Final purification involved dialysis against ultrapure water (MWCO: 3.5 kDa) for 12 h to isolate the assembled nanoparticles. For the synthesis of Cy5.5-labeled variants (Cy5.5@RBNPs), the dye was introduced at a 10% mass ratio during the primary reaction step, with all other parameters remaining unchanged.

### 2.4 Characterization of XDNPs and RBNPs

We characterized the chemical constituents of XDNPs employing an Agilent 1290 Infinity LC UHPLC system coupled to an AB Sciex TripleTOF 6600 mass spectrometer. For the RBNP formulation, drug loading efficiency (DLE) was calculated via HPLC analysis on a Thermo Scientific UltiMate 3000. Transmission electron microscopy (TEM) was performed using a Hitachi HT7800 microscope operating at 80 kV. For specimen preparation, the sample powder was dispersed in ddH_2_O by ultrasonication to form a homogeneous suspension. An aliquot (10 μL) of the suspension was then deposited onto a carbon-coated 200-mesh copper grid and dried at ambient temperature before observation. Additionally, we determined hydrodynamic size and surface charge (zeta potential) via dynamic light scattering (DLS) on a Malvern Zetasizer Nano S90; samples were prepared in aqueous suspension (1 mg/mL, pH 7.4) and bath-sonicated for 10 min before reading. Finally, spectral properties were confirmed using a PerkinElmer FTIR Two spectrometer and a Thermo Scientific NanoDrop One for UV-vis absorption.

### 2.5 *In vitro* release study of BBR from RBNPs

The *in vitro* release of BBR from RBNPs was assessed using a previously reported method with slight modifications [37]. Briefly, 20 mg of RBNPs was dispersed in SGF or SIF and incubated at 37 °C under continuous agitation. At designated time points, an aliquot of the release medium was collected for analysis, and an equal volume of fresh, pre-warmed corresponding buffer was immediately added to maintain sink conditions. The concentration of released BBR in the supernatant was determined by measuring the absorbance at 350 nm (OD_350_) using a Thermo Scientific NanoDrop One spectrophotometer, and calculated against a pre-established standard calibration curve.

### 2.6 Therapeutic efficacy of XDNPs and RBNPs in DSS-induced acute colitis mice

Male C57BL/6 mice, aged 6 to 8 weeks, were randomized into seven experimental arms (*n* = 8) after seven days of acclimatization. Groups included a vehicle control, a DSS model group, and treatment arms receiving 5-ASA, XXD, XDNPs, or RBNP at low (RBNPL) and high (RBNPH) doses. To induce colitis, animals consumed water containing 3% (w/v) dextran sulfate sodium (DSS) for ten consecutive days. Drug administration occurred via oral gavage between days 3 and 9, with dosages set at 100 mg/kg/day for 5-ASA and RBNPH, and 20 mg/kg/day for the remaining treatment groups. We monitored physiological status daily via body mass and disease activity index (DAI) scoring. At the endpoint (day 10), mice were euthanized to obtain colon samples for analysis and major organs (heart, liver, spleen, lung, kidney) for toxicity assessment. Feces were also collected and frozen at −80 °C for microbiome profiling. All procedures complied with the ethical guidelines of the China Academy of Chinese Medical Sciences (Approval No. 2025B220).

### 2.7 *In vivo* biodistribution imaging

To evaluate the biodistribution profile of RBNPs, colitis-bearing mice were randomly assigned to receive a single oral dose of either Cy5.5@RBNPs or a free mixture containing equivalent amounts of Cy5.5, BBR, and rhein (designated as the “Cy5.5” group). At predetermined time points (3, 6, 12, and 24 h) post-administration, three mice per group were anesthetized and subjected to whole-body fluorescence imaging using an IVIS Lumina III system (PerkinElmer). Following *in vivo* imaging, the mice were euthanized, and major organs (including heart, liver, spleen, lungs, kidneys) as well as the entire intestinal tract were collected for *ex vivo* fluorescence imaging to assess tissue-specific accumulation.

### 2.8 Enzyme-linked immunosorbent assay (ELISA)

We assessed *in vivo* colonic inflammation by determining the serum abundance of IL-1β, IL-6, and TNF-α via enzyme-linked immunosorbent assays (ELISA) following manufacturer guidelines. In parallel *in vitro* studies, RAW264.7 cells were cultured in 6-well plates for 24 h prior to experimentation. Macrophages were then subjected to co-incubation with LPS (1 μg/mL) and the specific therapeutic agents (XXD, XDNPs, or RBNPs at 20 μg/mL) for a 6 h period.

### 2.9 Microbiome analysis

Fecal microbiome analysis was performed via 16 S rRNA gene sequencing as previously described [14]. Microbial analysis relied on 16 S rRNA gene sequencing of DNA extracted via the QIAamp Fast DNA Stool Mini Kit (Qiagen). We specifically targeted the V4 domain for PCR amplification using custom-modified degenerate primers. Following electrophoretic separation and purification (Qiagen), the resulting amplicons were quantified and sequenced using the Illumina MiSeq system. In the bioinformatic phase, ‘Effective Tags' were processed in QIIME2, using DADA2 or Deblur for noise reduction to yield Amplicon Sequence Variants (ASVs). The Silva database served as the reference for taxonomic annotation. Furthermore, alpha diversity metrics (Shannon, Chao1, Observed_otus) were computed to characterize community richness and evenness, utilizing the computational resources of the Novogene and Oebiotech Cloud Platforms.

### 2.10 Cytotoxicity assay

Murine RAW264.7 macrophage-like cells were maintained in Dulbecco's Modified Eagle Medium (DMEM) enriched with 10% fetal bovine serum (FBS), under standard conditions (37 °C, 5% CO_2_, humidified atmosphere). For cytotoxicity assays, we plated the cells in 96-well formats at a concentration of 8000 cells per well, allowing a 24 h attachment period. Following this, the cultures were exposed to graded doses of XXD, XDNPs, or RBNPs for a subsequent 24 h. We finally evaluated cellular viability utilizing the Cell Counting Kit-8 (CCK-8) strictly adhering to the supplier's guidelines.

### 2.11 Cellular uptake

To evaluate cellular internalization kinetics, RAW264.7 macrophages were plated in 6-well dishes at a concentration of 2 × 10^5^ cells per well. We subsequently exposed the cultures to DMEM supplemented with either Cy5.5@RBNPs (2.2 μg/mL) or an equivalent concentration of free Cy5.5 (0.22 μg/mL) for defined intervals of 0, 1, 3, and 6 h. Following the incubation period, samples underwent three washes with PBS to eliminate extracellular dye. Finally, cells were collected and subjected to quantitative analysis using a Beckman Coulter flow cytometer.

### 2.12 Macrophage polarization assay

Following a 24 h attachment period in 6-well plates, RAW264.7 macrophages were challenged with LPS (1 μg/mL) for 6 h, either alone or in combination with XXD, XDNPs, or RBNPs. Upon completion of the treatment, we rinsed the cultures three times using PBS and dispersed the resulting pellets in100 μL of buffer, Immunostaining was conducted by incubating the cells for 30 min in the dark with antibodies targeting F4/80 (1 μg/test), CD86 (1 μg/test), and CD206 (0.5 μg/test). Finally, samples underwent three additional PBS washes before being resuspended in 500 μL PBS for flow cytometric evaluation.

### 2.13 Assessment of reactive oxygen species

Intracellular accumulation of ROS was visualized and quantified through fluorescence microscopy and flow cytometry. Briefly, 2 × 10^5^ RAW264.7 cells were inoculated into 6-well plates and cultured for 24 h. The medium was then replaced with LPS (1 μg/mL) supplemented with or without XXD, XDNPs, or RBNPs for a 6 h duration. To detect ROS, cells were rinsed thrice with PBS and incubated with 5 μM DCFH-DA (37 °C, 30 min) in the absence of light. Upon completion of staining, samples were washed, suspended in 500 μL PBS, and subjected to analysis. Concurrently, levels of nitric oxide (NO) and hydrogen peroxide (H_2_O_2_) were determined using commercially available kits as per the manufacturer's recommendations.

### 2.14 RNA extraction and real-time PCR

Total RNA extraction from tissue homogenates and cell lysates was executed via the TRIzol method (TransGen Biotech). We generated cDNA from these templates using a commercial reverse transcription kit. Subsequent gene expression analysis employed SYBR Green-based real-time PCR. The reaction program comprised a pre-incubation at 95 °C for 5 min, f and then 40 cycles of denaturation at 95 °C for 10 s combined with annealing at 60 °C (30 s). All primer designs are provided in Table S1.

### 2.15 Western blotting

Total protein lysates were generated using RIPA buffer containing a cocktail of protease and phosphatase inhibitors. For Western blot analysis of colonic tissues, total proteins were extracted from six randomly selected mice per group. To minimize inter-individual biological variability, equal amounts of protein from each sample were pooled prior to immunoblotting. All experiments were performed with three technical replicates to ensure data reproducibility. Equalized protein loads were resolved via SDS-PAGE and immobilized onto PVDF membranes with a 0.22 μm PVDF membranes. Pore size. Membranes underwent blocking with 5% non-fat dry milk for 1 h prior to probing with specific primary antibodies overnight (4 °C). Subsequently, membranes were washed in TBST and incubated with HRP-conjugated secondary antibodies for 60 min. Final detection was achieved using an Azure 600 system, with band intensity quantified via ImageJ software.

### 2.16 Activity-based protein profiling

ABPP was performed following previously described protocols with minor modifications [38]. *In situ* cellular labeling was initiated by incubating RAW264.7 cultures with berberine probe (BBR-P) for 4 h. Unbound probe was removed via PBS washing, followed by irradiation at 365 nm at 4 °C 10 min to covalently link targets. Lysis was carried out in a 0.1% Triton X-100/PBS solution containing protease inhibitors. The resulting lysate underwent copper-catalyzed azide-alkyne cycloaddition (CuAAC) with 50 μM TAMRA-PEG_3_-N_3_, 100 μM TBTA, 1 mM TCEP, and 1 mM copper sulfate for 1 h at 29 °C. Following acetone precipitation and boiling in SDS buffer, samples were resolved by 12% electrophoresis and imaged for fluorescence. For affinity enrichment, the click reaction employed Biotin-PEG_3_-N_3_ labeled proteins were captured on agarose beads and eluted for proteomic analysis (LC-MS/MS).

### 2.17 LC-MS/MS analysis and data processing

Reduction and alkylation of biotinylated proteins were achieved using 10 mM DTT and 25 mM iodoacetamide, respectively, followed by enzymatic digestion with trypsin at 37 °C for 17 h. TMT 10plex reagents were employed for peptide labeling according to standard protocols. Analytical separation and detection utilized an UltiMate 3000 nano-LC linked to an Orbitrap Fusion Lumos. For data analysis, raw files were imported into Proteome Discoverer 2.4. The Sequest HT algorithm facilitated searching against the *Mus musculus* proteome (UniProtKB, Jan 2024), allowing for two missed cleavages and a minimum peptide length of seven amino acids. Mass tolerances were fixed at 10 ppm (MS1) and 0.02 Da (MS2). Modifications included static carbamidomethylation and variable oxidation/acetylation. Valid identifications required a false discovery rate (FDR) < 1% as determined by Percolator, with quantification derived from reporter ion data.

### 2.18 Protein target labeling by BBR probe

To confirm target engagement *in situ*, RAW264.7 cells were co-incubated with BBR-P (50 μM) and an excess of free BBR (200 μM) for 4 h. Following UV irradiation and click chemistry, cell lysates were incubated with NeutrAvidin agarose resin to enrich labeled proteins, which were subsequently analyzed by Western blotting. To determine whether VDAC1 is a direct binding target of BBR, *in vitro* assays were conducted using recombinant human VDAC1 (rhVDAC1). Briefly, rhVDAC1 (2.5 μg) was dissolved in 50 μL PBS and incubated at 25 °C for 2 h with either DMSO (vehicle), BBR-P (50 μM), or BBR-P (50 μM) in the presence of excess BBR (200 μM). After incubation, samples underwent a click reaction with TAMRA-PEG_3_-N_3_ and were analyzed by in-gel fluorescence scanning and SDS-PAGE.

### 2.19 Cellular thermal shift assay (CETSA)

Cellular thermal shift assays (CETSA) were conducted by treating RAW264.7 cells with 20 μM BBR or equivalent DMSO for 4 h. After washing twice, cells were collected and partitioned into eight tubes containing phosphatase and protease inhibitors. Thermal shock was applied for 3 min at designated temperatures utilizing a thermal cycler, followed by immediate ice incubation. Lysis involved sonication in PBS with 0.1%Triton X-100/PBS. We recovered soluble supernatants by centrifuging at 13,000 rcf at 4 °C for 30 min. Samples were then prepared with 5 × SDS-PAGE loading buffer, heat-denatured, and analyzed via Western blotting.

### 2.20 Measurement of cytosolic mtDNA

Cytosolic mtDNA leakage was assessed by treating RAW264.7 cells with either 10 μM BBR or 20 μg/mL XXD, XDNPs, or RBNPs. Fractionation followed referenced methods [39,40], involving PBS washing and resuspension in inhibitor-supplemented mitochondrial buffer. We sonicated the samples and centrifuged at 11,000 rcf for 10 min at 4 °C to pellet insoluble organelles. The clarified supernatant served as the source for cytosolic DNA, which was isolated via a commercial kit and quantified using gene-specific qPCR.

### 2.21 siRNA transfection

We induced VDAC1 knockdown in RAW264.7 cells via transfection with 20 nM specific siRNA (siVDAC1) versus negative control. Delivery was facilitated by Lipofectamine 2000 reagent (Thermo Fisher Scientific), adhering to the standard manufacturer instructions. The siVDAC1 sequences were as follows: Forward, 5′-GGCUAUAAGACGAUGAAUTT-3′; Reverse, 5′-AUUCAUCCGUCUUAUAGCCTT-3′.

### 2.22 Statistical analysis

Quantitative data are expressed as the mean ± standard deviation (SD). Statistical analyses were performed using one-way analysis of variance (ANOVA) followed by Tukey post hoc tests. All statistical computations were conducted using GraphPad Prism 8.0 software (GraphPad Software, USA), and a p-value <0.05 was considered statistically significant.

## 3 Results

### 3.1 Preparation and characterization of self-assembled nanoparticles derived from Xiexin Decoction

**Fig. 1:**
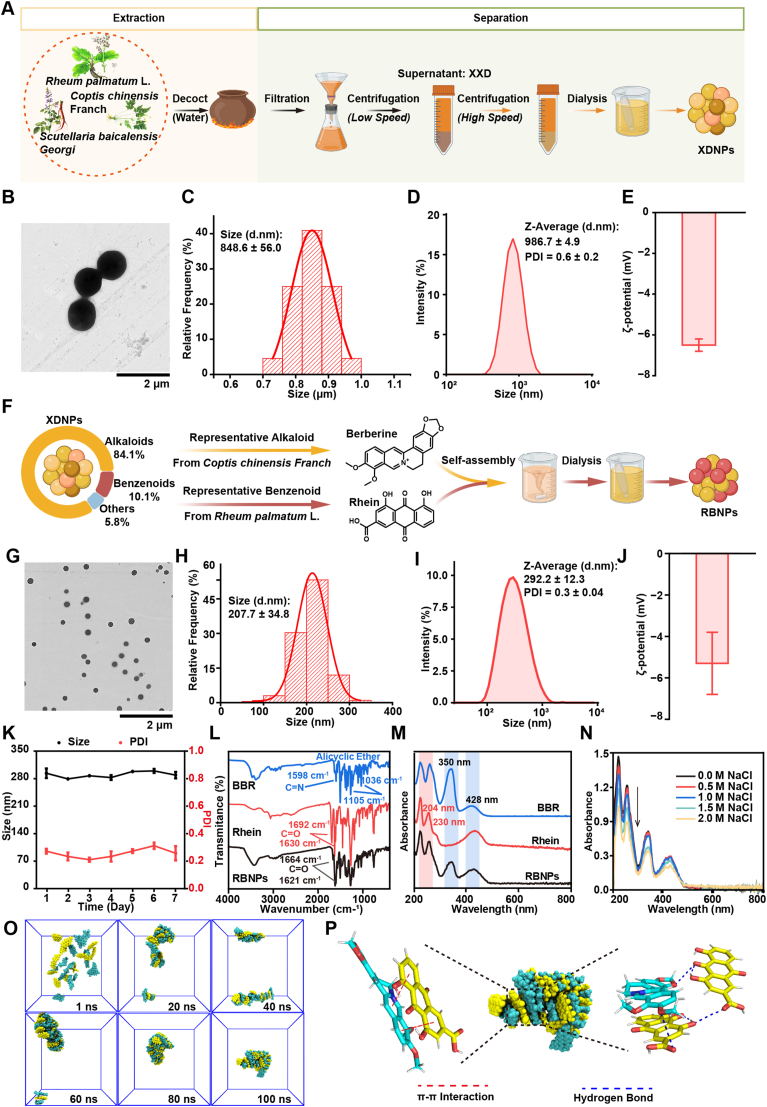
Preparation and Characterization of Self-Assembled Nanoparticles Derived from Xiexin Decoction. **(A)** Schematic illustration of the separation and extraction process for XDNPs from XXD. **(B)** Transmission electron microscopy (TEM) image showing the morphology of XDNPs. **(C)** Size distribution of XDNPs quantified from at least 200 nanoparticles in the TEM image. **(D)** Hydrodynamic size distribution and polydispersity index (PDI) of XDNPs (*n* = 3). **(E)** Zeta potential of XDNPs (*n* = 3). **(F)** Schematic illustration of the self-assembly of BBR and rhein to form RBNPs. **(G)** TEM image showing the morphology of RBNPs. **(H)** Size distribution of RBNPs quantified from at least 200 nanoparticles in the TEM image. **(I)** Hydrodynamic size distribution and PDI of RBNPs (*n* = 3). **(J)** Zeta potential of RBNPs (*n* = 3). **(K)** Colloidal stability of RBNPs, assessed by monitoring hydrodynamic size and PDI over a 7-day period (*n* = 3). **(L)** Fourier-transform infrared (FT-IR) spectra of RBNPs, free BBR, and free rhein (*n* = 3). **(M)** Ultraviolet-visible (UV-Vis) absorption spectra of RBNPs, free BBR, and free rhein (*n* = 3). **(N)** UV-Vis absorption spectra of RBNPs dispersed in NaCl solutions of varying concentrations (*n* = 3). **(O)** Molecular dynamics simulation of BBR and rhein self-assembly over 0–100 ns (BBR: green; rhein: yellow). **(P)** 3D force interaction diagrams of BBR (green) and rhein (yellow), showing hydrophobic interactions (blue dotted lines) and π–π stacking (red dotted lines).

To investigate the intrinsic nanostructures formed within the traditional Chinese medicine XXD, an aqueous extract was prepared from a standardized mixture (2:1:1, w/w) of *Rheum palmatum* L., *Coptis chinensis* Franch., and *Scutellaria baicalensis* Georgi using conventional decoction procedures. After filtration and low-speed centrifugation to remove insoluble residues, the supernatant constituted the crude XXD extract. Naturally occurring, self-assembled nanoparticles (XDNPs) were subsequently isolated through sequential centrifugation and dialysis to eliminate large aggregates and free small molecules (Fig. 1A). TEM revealed that XDNPs exhibited a uniform spherical morphology (Fig. 1B), with an average diameter of 848.6 ± 56.0 nm (Fig. 1C). Dynamic light scattering (DLS) analysis in aqueous medium showed a hydrodynamic diameter of 986.7 ± 4.9 nm and a polydispersity index (PDI) of 0.6 ± 0.2, indicating a relatively broad size distribution (Fig. 1D). The zeta potential was measured as −6.5 ± 0.3 mV (Fig. 1E), suggesting moderate colloidal stability attributed to surface negative charge. Collectively, these findings demonstrate that bioactive constituents within XXD spontaneously self-assemble into stable nanoscale structures during the decoction process. Compositional analysis of XDNPs by ultra-high-performance liquid chromatography–quadrupole time-of-flight mass spectrometry (UHPLC-QTOF-MS) identified alkaloids (84.1%) and benzenoids (10.1%) as the predominant chemical classes (Fig. 1F and Fig. S1).

To elucidate the molecular interactions responsible for nanoparticle formation, two representative components were selected: BBR, a major alkaloid from *Coptis chinensis*, and rhein, a key benzenoid from *Rheum palmatum*. Based on the hypothesis that BBR and rhein are critical drivers of nanoparticle assembly, binary, carrier-free nanoparticles (RBNPs) were synthesized via a nanoprecipitation approach leveraging their inherent intermolecular interactions (Fig. 1F). The as-prepared RBNPs exhibited a uniform spherical morphology under TEM with an average diameter of 207.0 ± 34.8 nm (Fig. 1G and H). DLS analysis revealed a hydrodynamic diameter of 292.2 ± 12.3 nm, a PDI of 0.30 ± 0.04, and a zeta potential of −5.3 ± 1.5 mV (Fig. 1I and J). RBNPs remained stable in aqueous solution at 37 °C for at least one week, as indicated by negligible changes in hydrodynamic diameter and PDI (Fig. 1K). Spectroscopic analyses further elucidated the intermolecular forces stabilizing RBNPs. Fourier transform infrared (FTIR) spectroscopy revealed noncovalent interactions between BBR and rhein. Characteristic BBR peaks were observed at 1598 cm^−1^ (C=N stretch) and 1105–1036 cm^−1^ (alicyclic ether vibrations), whereas rhein exhibited C=O and O–H stretching bands at 1692 and 1630 cm^−1^, respectively (Fig. 1L). Upon RBNP formation, the C=O stretching of rhein shifted to 1664 and 1621 cm^−1^, indicative of hydrogen bonding and π–π stacking interactions between the carbonyl groups of rhein and the aromatic system of BBR [41]. Supporting this, UV-Vis spectra confirmed that both BBR and rhein absorption peaks were retained within the RBNPs, validating their co-assembly into a unified nanosystem (Fig. 1M). Furthermore, increasing NaCl concentrations resulted in a concentration-dependent reduction in the turbidity of RBNP suspensions (Fig. 1N), suggesting that electrostatic interactions play a pivotal role in nanoparticle stabilization [42]. Molecular dynamics simulations showed that BBR molecules encapsulate rhein through hydrophobic interactions and hydrogen bonding. The radial distribution function analysis demonstrated close packing between the nitrogen atoms of BBR and the oxygen atoms of rhein (Fig. 1O). The aromatic rings of both molecules promote π–π stacking, while hydroxyl groups contribute to hydrogen bond formation (Fig. 1P). Root mean square deviation (RMSD) trajectories indicated that the system rapidly reached equilibrium (Fig. S2A), and subsequent decreases in solvent-accessible surface area (SASA) and radius of gyration (Rg) beyond 20 ns suggested a transition toward a compact, aggregated conformation consistent with stable self-assembly (Fig. S2B–C). Taken together, both the naturally derived XDNPs from XXD and the synthetically assembled RBNPs composed of BBR and rhein were successfully prepared and characterized as stable nanoscale entities. The combined spectroscopic and computational evidence highlights that electrostatic attraction, hydrogen bonding, and π–π stacking collectively govern the self-assembly of RBNPs, providing a mechanistic model for understanding the formation of natural nanocomponents in XXD.

### 3.2 Self-assembled nanoparticles exhibit gastrointestinal stability and prolonged colonic retention in UC mice

**Fig. 2:**
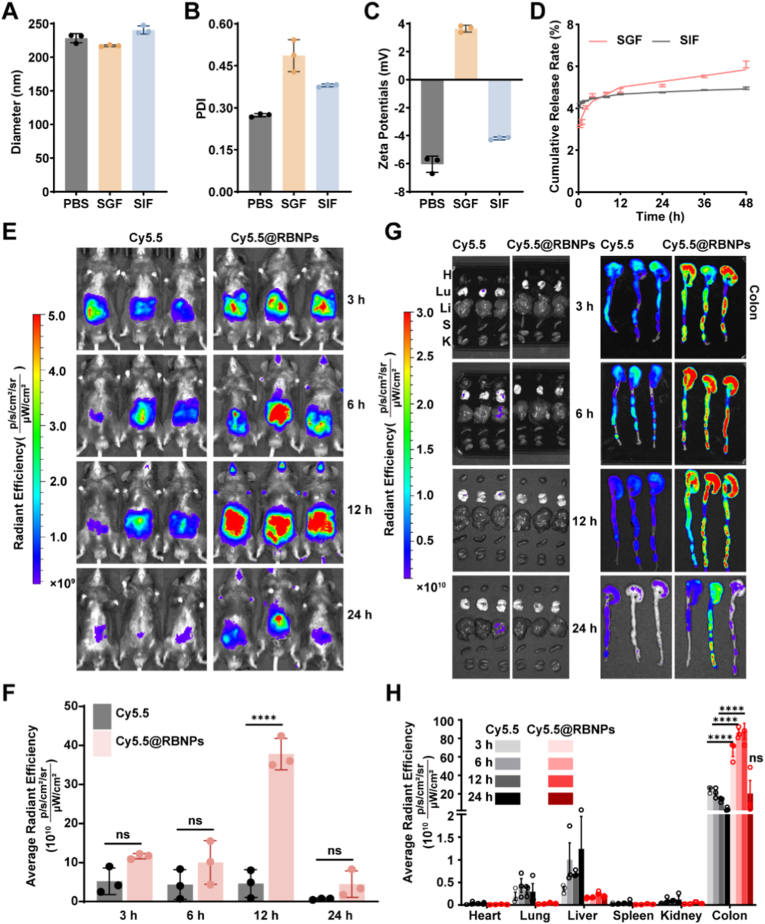
Gastrointestinal stability and biodistribution of RBNPs. **(A**–**C)** Hydrodynamic diameter (A), PDI (B), and zeta potential (C) of RBNPs incubated in PBS, SGF, or SIF. **(D)***In vitro* cumulative release profiles of BBR from RBNPs in SGF and SIF over 48 h. **(E**–**F)** Representative *in vivo* fluorescence images (E) and quantification of radiant efficiency (F) in mice following oral administration of Cy5.5@RBNPs or free Cy5.5. **(G)***Ex vivo* fluorescence images of major organs (H, heart; Lu, lung; Li, liver; S, spleen; K, kidney) and colons excised at indicated time points. **(H)** Quantification of radiant efficiency in major organs and colons. Data are presented as mean ± SD (*n* = 3). Statistical significance was assessed by one-way ANOVA with Tukey's post hoc test, ∗∗∗∗*p* < 0.0001; ns, not significant.

To assess the suitability of RBNPs for oral administration, we evaluated their stability and release profiles in simulated gastric fluid (SGF, pH 1.2) and simulated intestinal fluid (SIF, pH 6.8). As shown in Fig. 2A, RBNPs maintained a stable hydrodynamic diameter and morphology in both media, demonstrating structural integrity under gastrointestinal conditions. Compared to PBS, a slight increase in PDI was observed in SGF and SIF (Fig. 2B), likely attributable to minor fluctuations in dispersion uniformity due to the complex ionic and proteinaceous composition of the simulated fluids. Correspondingly, the zeta potential of RBNPs shifted in response to changes in pH and ionic strength (e.g., from −6.0 mV in PBS to −4.2 mV in SIF and +3.6 mV in SGF, Fig. 2C). *In vitro* release assays further confirmed that RBNPs effectively prevented premature drug leakage, exhibiting sustained-release characteristics over 48 h with cumulative BBR release of approximately 6% in SGF and 4% in SIF (Fig. 2D). These results demonstrate that RBNPs resist gastric degradation and minimize drug loss during upper gastrointestinal transit, ensuring efficient delivery of the therapeutic payload to the intestinal tract.

To visualize the targeting capability of RBNPs to the inflamed colon, *in vivo* fluorescence imaging was performed following oral administration of Cy5.5@RBNPs to UC mice. As shown in Fig. 2E and F, the free Cy5.5 mixture group exhibited weak and rapidly declining fluorescence signals, indicating rapid systemic absorption and clearance. In contrast, the Cy5.5@RBNPs group demonstrated significantly enhanced retention and accumulation within the gastrointestinal tract, with average fluorescence intensity consistently exceeding that of the free control group at 3, 6, 12, and 24 h post-administration. *Ex vivo* imaging of dissected tissues (Fig. 2G) revealed that RBNPs rapidly accumulated in colon tissue and exhibited prolonged retention, whereas the free Cy5.5 mixture showed rapid signal attenuation in the colon followed by gradual migration to the liver. Quantitative analysis (Fig. 2H) confirmed that RBNPs exhibited significantly higher colonic affinity and extended residence time compared to the free control. This enhanced accumulation and reduced clearance at the inflamed site are critical for achieving localized therapeutic effects in UC.

### 3.3 Self-assembled nanoparticles attenuate disease severity in DSS-induced colitis mice

**Fig. 3:**
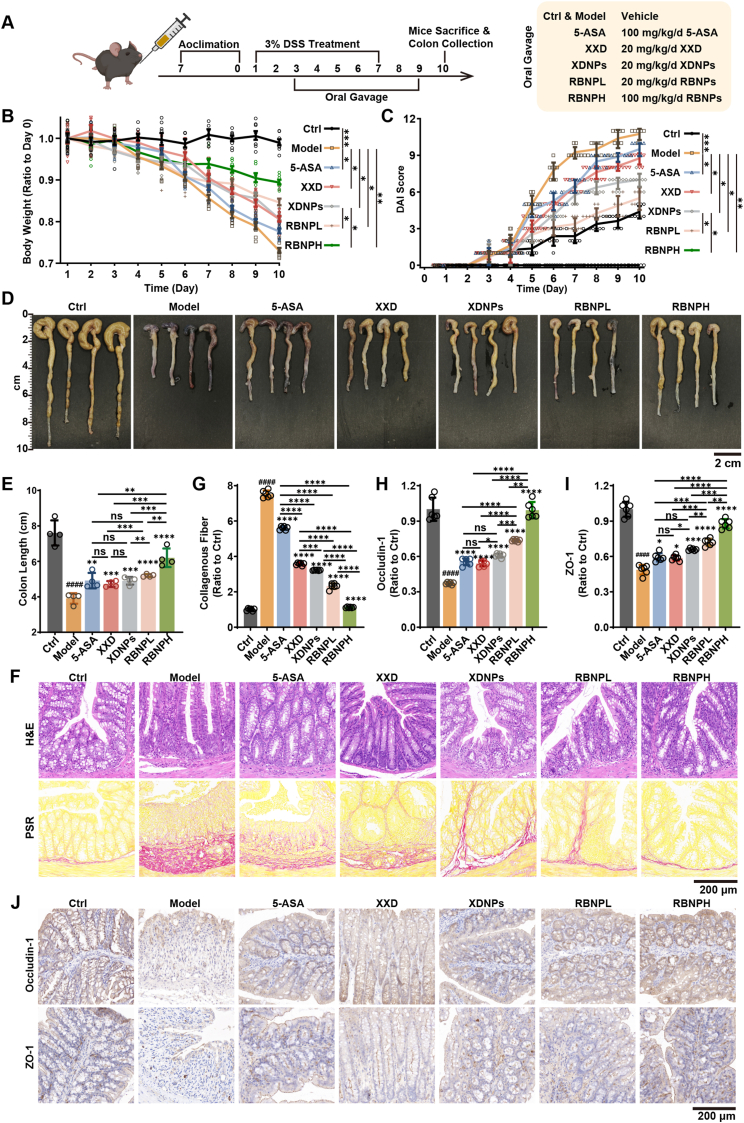
Therapeutic Effects of XXD, XDNPs, and RBNPs in DSS-Induced Colitis in Mice. **(A)** Schematic illustration of the experimental design and treatment schedule. **(B**–**C)** Changes in body weight (*n* = 8) (B) and disease activity index (DAI, *n* = 8) (C) over the course of the study. **(D)** Representative images of excised colons from each treatment group at the study endpoint. **(E)** Quantification of colon length across groups (*n* = 4). **(F)** Representative histological images of colonic sections stained with hematoxylin and eosin (H&E) or PicroSirius Red (PSR). **(G)** Quantitative analysis of collagen deposition from PSR-stained sections (*n* = 6). **(H**–**I)** Immunohistochemical quantification of Occludin (H) and ZO-1 (I) protein expression levels (*n* = 6). **(J)** Representative photomicrographs show the staining for Occludin and ZO-1 in colonic tissue sections from each experimental group. Data are presented as mean ± SD. Statistical significance was determined using one-way ANOVA followed by Tukey's post hoc test. ∗*p* < 0.05, ∗∗*p* < 0.01, ∗∗∗*p* < 0.001, ∗∗∗∗*p* < 0.0001 versus Model group; ^####^*p* < 0.0001 versus Ctrl group.

Building on the documented clinical efficacy of XXD in UC, we evaluated the therapeutic potential of both naturally derived (XDNPs) and synthetic (RBNPs) nanoparticles in a dextran sulfate sodium (DSS)-induced acute colitis mouse model, as illustrated in the experimental schematic (Fig. 3A). 5-Aminosalicylic acid (5-ASA), a first-line clinical therapy for UC, served as a positive control. Mice in the Model group exhibited significant body weight loss and elevated DAI compared with the control (Ctrl) group, confirming successful induction of colitis (Fig. 3B and C). Treatment with XDNPs and RBNPs, at both low and high doses, markedly attenuated weight loss and reduced DAI scores throughout the experimental period. Notably, the protective effects of XDNPs and RBNPs were significantly superior to those observed with the crude XXD extract or 5-ASA. DSS administration also induced pronounced colon shortening, a hallmark of colonic inflammation and tissue damage. Consistent with the clinical parameters, XDNPs and RBNPs preserved colon length, whereas 5-ASA and XXD produced only modest improvements (Fig. 3D and E).

Histopathological analyses further confirmed the enhanced therapeutic efficacy of the nanoparticles. Hematoxylin and eosin (H&E) staining of colon sections from the Model group revealed severe mucosal injury, including extensive epithelial necrosis, crypt distortion, and dense inflammatory cell infiltration (Fig. 3F). In contrast, XDNPs and RBNPs, particularly high-dose RBNPs (RBNPH), substantially ameliorated these pathological changes, with colonic architecture largely preserved and comparable to the healthy Ctrl group. Considering that chronic UC can lead to fibrosis, Picrosirius red (PSR) staining was performed to evaluate collagen deposition. As expected, the Model group exhibited marked collagen accumulation. While XXD, XDNPs, and low-dose RBNPs (RBNPL) partially reduced collagen deposition, only RBNPH treatment effectively prevented fibrotic changes, achieving near-complete restoration to Ctrl levels (Fig. 3F and G). Maintenance of intestinal barrier integrity is critical for UC management. Immunofluorescence staining of key tight junction proteins, Occludin-1 and Zonula Occludens-1 (ZO-1), revealed substantial disruption and loss at the epithelial junctions in the Model group. Treatment with XDNPs and RBNPs restored both the expression and membrane localization of these proteins to levels comparable with the Ctrl group (Fig. 3H–J). This restoration provides a mechanistic basis for the observed reduction in inflammation and tissue injury. Collectively, these *in vivo* findings demonstrate that both naturally derived XDNPs and synthetically assembled RBNPs confer robust protection against DSS-induced colitis. Their therapeutic efficacy exceeds that of the crude XXD decoction and 5-ASA, as evidenced by superior mitigation of clinical symptoms, preservation of colon morphology, inhibition of fibrosis, and restoration of intestinal barrier integrity.

### 3.4 Self-assembled nanoparticles potently suppress inflammatory responses in colitis

**Fig. 4:**
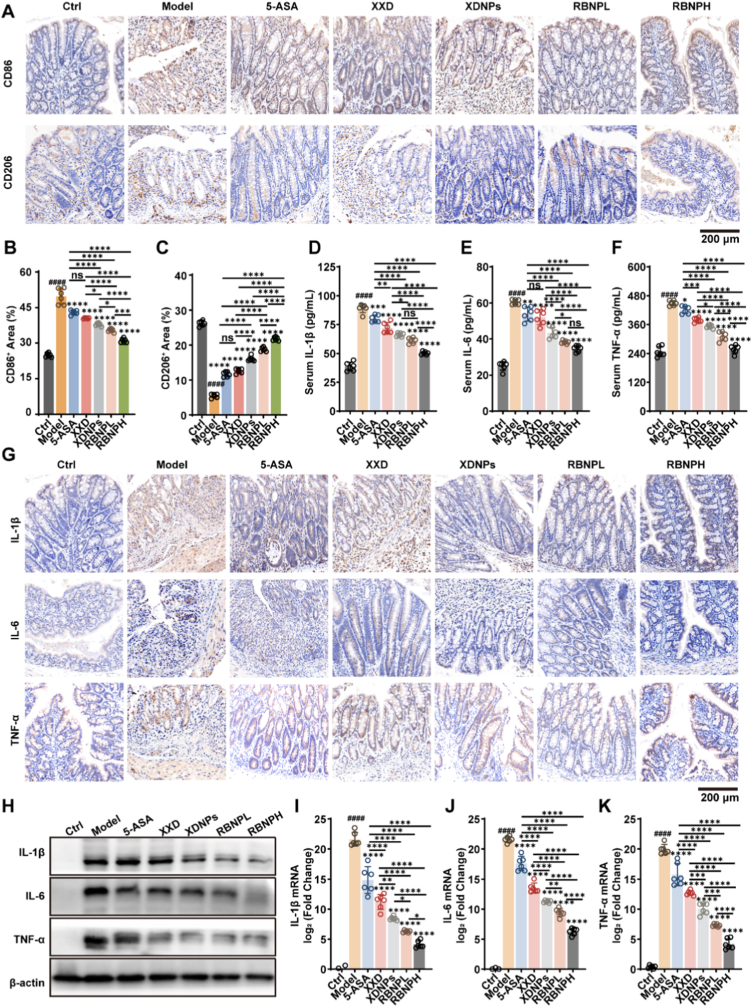
XDNPs and RBNPs attenuate inflammatory responses in DSS-induced colitis. **(A)** Representative immunohistochemical (IHC) images of colon sections stained for macrophage markers CD86 (M1) and CD206 (M2). **(B**–**C)** Quantitative analysis of CD86-positive (B) and CD206-positive (C) area fractions. **(D**–**F)** Serum levels of pro-inflammatory cytokines IL-1β (D), IL-6 (E), and TNF-α (F) measured by ELISA. **(G)** Representative IHC images of IL-1β, IL-6, and TNF-α expression in colon tissue. **(H)** Western blot analysis of IL-1β, IL-6, and TNF-α in colon tissue lysates (*n* = 6 mice per group). **(I**–**K)** Relative mRNA expression of IL-1β (I), IL-6 (J), and TNF-α (K) in colon tissues, determined by RT-qPCR. Data are presented as mean ± SD (*n* = 6). Statistical significance was determined using one-way ANOVA with Tukey's post hoc test. ∗*p* < 0.05, ∗∗*p* < 0.01, ∗∗∗*p* < 0.001, ∗∗∗∗*p* < 0.0001 versus Model group; ^####^*p* < 0.0001 versus Ctrl group.

UC is characterized by chronic, relapsing inflammation driven by dysregulated immune responses. Macrophages play a central role in maintaining intestinal immune homeostasis, and their aberrant polarization toward a pro-inflammatory M1 phenotype at the expense of the anti-inflammatory M2 phenotype is a hallmark of UC pathogenesis [39,40]. We therefore investigated the effects of XDNPs and RBNPs on macrophage polarization and downstream inflammatory cytokine production. In the DSS-induced colitis model, nanoparticle treatment significantly rectified macrophage polarization imbalances. Immunohistochemical (IHC) staining and statistical analysis revealed that, compared with the control group, the proportion of M1 macrophages (CD86^+^) in the colonic lamina propria monocytes was significantly elevated in the Model group, while the proportion of M2 macrophages (CD206^+^) was correspondingly reduced (Fig. 4A–C). Administration of XDNPs and RBNPs exerted a robust immunomodulatory effect, substantially reducing the frequency of pro-inflammatory M1 macrophages while enhancing the anti-inflammatory M2 population. This shift toward a reparative macrophage phenotype was significantly more pronounced with nanoparticle treatment than with either the conventional XXD extract or 5-ASA.

Consistent with the macrophage polarization results, nanoparticle treatment markedly suppressed systemic and local inflammatory cytokine production. ELISA analysis of serum samples showed significantly elevated levels of the key pro-inflammatory cytokines IL-1β, IL-6, and TNF-α in the Model group (Fig. 4D–F). All treatment groups demonstrated reductions in these cytokines; however, XDNPs and RBNPs elicited a substantially stronger suppressive effect compared to XXD alone. IHC staining (Fig. 4G and Fig. S3) and Western blot analysis (Fig. 4H and Fig. S4) of IL-1β, IL-6, and TNF-α in colonic tissues further confirmed that XDNPs and RBNPs effectively attenuated the DSS-induced elevation of pro-inflammatory cytokines, exhibiting superior efficacy compared to 5-ASA and XXD. Complementary qPCR analysis of colon tissues corroborated these findings, revealing significant upregulation of IL-1β, IL-6, and TNF-α mRNA in the Model group, which was effectively attenuated by XDNPs and RBNPs, restoring transcript levels toward those observed in the Ctrl group (Fig. 4I–K). Collectively, these results demonstrate that XDNPs and RBNPs exert potent anti-inflammatory effects in DSS-induced colitis, primarily by reprogramming macrophages toward an anti-inflammatory M2 phenotype and consequently suppressing critical pro-inflammatory cytokines both systemically and locally within the colon. The enhanced efficacy of the nanoparticle formulations underscores their advantage in delivering active components more efficiently than conventional decoctions.

### 3.5 Self-assembled nanoparticles restore gut microbiota homeostasis in colitis mice

**Fig. 5:**
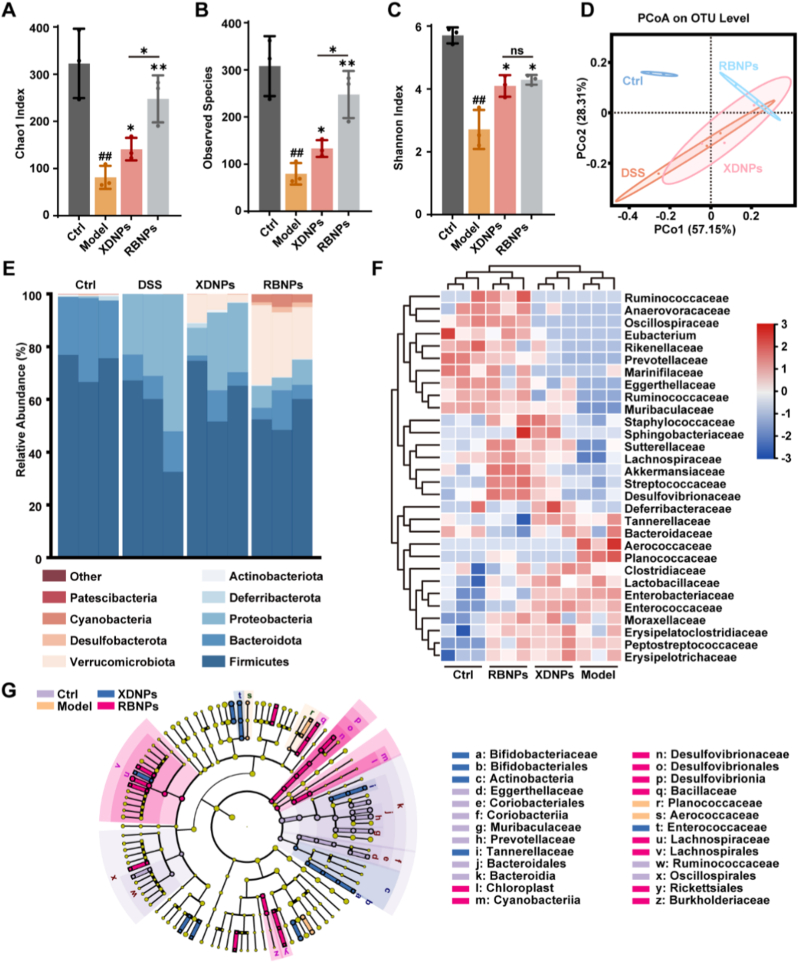
**XDNPs and RBNPs modulate gut microbiota homeostasis in DSS-induced colitis mice. (A**–**C)** Alpha diversity metrics, including **(A)** Chao1, **(B)** observed species, and **(C)** Shannon indices. **(D)** Principal Coordinates Analysis (PCoA) plot depicting beta diversity based on operational taxonomic unit (OTU) abundances. **(E)** Relative abundance of major microbial taxa at the phylum level. **(F)** Heatmap illustrating the relative abundance of the top 30 most abundant microbial families. **(G)** LEfSe-derived multilevel species-level phylogenetic tree highlighting differentially enriched taxa. Data are expressed as mean ± SD (*n* = 3). Statistical significance was determined using one-way ANOVA followed by Tukey's post hoc test. ∗*p* < 0.05 versus Model group; ^##^*p* < 0.01 versus Ctrl group.

Accumulating evidence implicates gut dysbiosis as a critical pathogenic factor in UC, typically characterized by reduced microbial diversity and a community composition biased toward pro-inflammatory taxa [43]. Restoration of a balanced gut microbiota represents a promising therapeutic strategy for UC [44,45]. We therefore investigated whether the beneficial effects of XDNPs and RBNPs involve modulation of gut microbiome. Fecal samples collected at the study endpoint were subjected to 16 S ribosomal RNA (rRNA) gene sequencing and subsequent bioinformatic analyses. Treatment with XDNPs and RBNPs effectively reversed DSS-induced reductions in microbial diversity. Alpha diversity indices, including Chao1 (richness), observed species (richness), and Shannon (diversity and evenness), were significantly decreased in the Model group relative to the healthy Ctrl group, confirming DSS-induced dysbiosis. Notably, administration of XDNPs and RBNPs significantly restored these indices toward Ctrl levels (Fig. 5A–C). Beyond diversity, nanoparticle treatment normalized overall microbial community structure. Principal coordinate analysis (PCoA) revealed distinct clustering patterns reflecting beta diversity. Microbial communities from the Model group were widely dispersed, indicating pronounced inter-individual variability and instability. In contrast, communities from RBNP-treated mice clustered closely with those of the Ctrl group, demonstrating a significant restoration of a healthy microbial profile (Fig. 5D).

At the phylum level, characteristic dysbiotic shifts observed in the Model group were attenuated by nanoparticle therapy. Bacteroidetes, Firmicutes, and Proteobacteria dominated the gut microbiota across all groups (Fig. 5E). DSS-induced colitis resulted in a decreased Firmicutes/Bacteroidetes (F/B) ratio, a recognized marker of gut dysbiosis in inflammatory bowel disease [43,46], alongside a notable expansion of Proteobacteria, which includes multiple opportunistic pathogens such as *Escherichia* and *Salmonella*. Treatment with XDNPs and RBNPs partially normalized the F/B ratio and reduced the relative abundance of Proteobacteria. Genus-level heatmap analysis further highlighted taxa selectively modulated by nanoparticle treatment (Fig. 5F). XDNPs and RBNPs significantly increased the abundance of beneficial bacterial families associated with gut health and anti-inflammatory functions, including *Ruminococcaceae*, *Muribaculaceae*, and *Lachnospiraceae*. These bacteria contribute to intestinal barrier integrity, immune regulation, and metabolic homeostasis through the production of short-chain fatty acids (SCFAs) [47], [48], [49] [[47], [48], [49]]. Conversely, the Model group exhibited enrichment of *Enterobacteriaceae*, a family strongly linked to gut inflammation and dysbiosis [50]. Nanoparticle treatment significantly suppressed this pathological expansion. To identify taxa most discriminative treatment effects, Linear Discriminant Analysis Effect Size (LEfSe) was performed (Fig. 5G and Fig. S5). This analysis confirmed significant enrichment of health-promoting lineages, including *Lachnospirales* and *Bifidobacteriales*, in the XDNPs and RBNPs groups. *Bifidobacteriales* contribute to vitamin and SCFA production, while *Lachnospiraceae* are major butyrate producers, supporting colonocyte function, modulating immune responses, and exerting anti-inflammatory effects [51,52]. Collectively, these microbiome analyses demonstrate that XDNPs and RBNPs profoundly restore gut microbial homeostasis in DSS-induced colitis. The re-establishment of a microbiota enriched in barrier-supporting, anti-inflammatory, and health-promoting taxa provides a compelling mechanistic basis for the observed attenuation of intestinal inflammation and improvement of epithelial barrier integrity following nanoparticle treatment.

### 3.6 Proteomic profiling of colonic tissue elucidates the therapeutic effects and molecular mechanisms of RBNPs and XDNPs

**Fig. 6:**
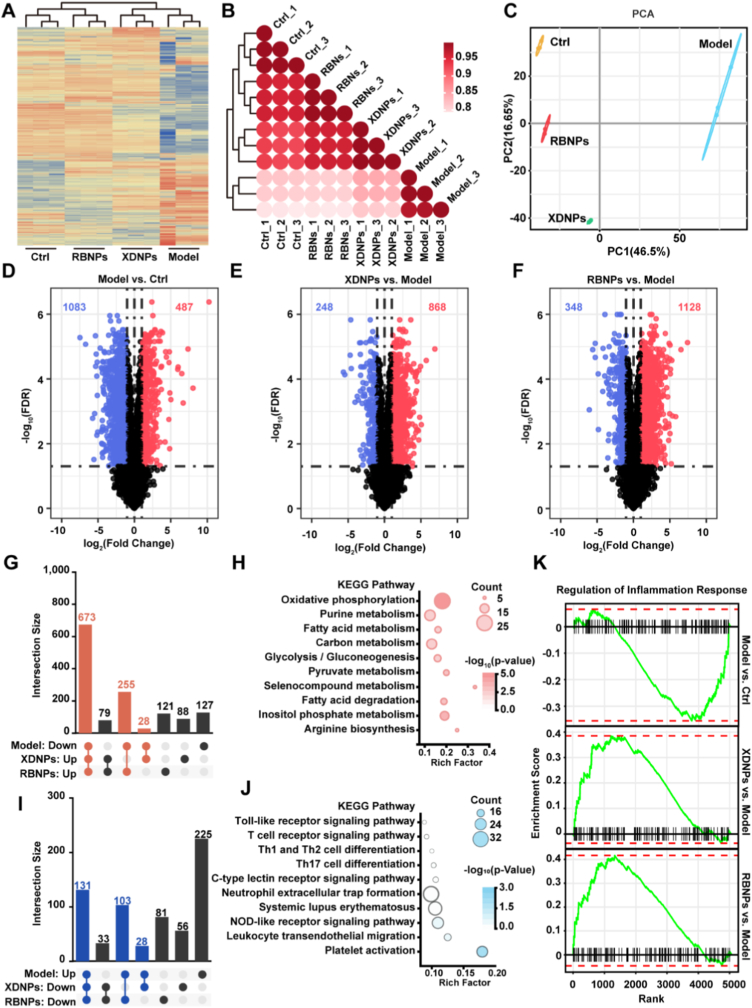
XDNPs and RBNPs restore DSS-induced proteomic alterations by regulating metabolic and inflammatory pathways. **(A)** Heatmap of all quantified proteins across the Ctrl, Model, XDNPs and RBNPs groups. **(B)** Pearson correlation heatmap depicting inter-sample relationships based on proteomic profiles. **(C)** Principal Component Analysis (PCA) showing the distribution and separation of samples from different groups. **(D**–**F)** Volcano plots illustrating differentially expressed proteins (DEPs) between indicated comparison groups. Red dots denote significantly upregulated proteins, and blue dots denote significantly downregulated proteins (FDR <0.05, |log_2_(Fold Change)| > 1). **(G)** The UpSet plot illustrating the overlap between proteins significantly downregulated by DSS and those significantly up recovered following XDNPs and RBNPs treatment. **(H)** KEGG enrichment analysis of recovery proteins co-upregulated by XDNPs and RBNPs treatment. **(I)** The UpSet plot illustrating the overlap between proteins significantly upregulated by DSS and those significantly down recovered following XDNPs and RBNPs treatment. **(J)** KEGG enrichment analysis of recovery proteins co-downregulated by XDNPs and RBNPs treatment. **(K)** Gene Set Enrichment Analysis (GSEA) plots for the “Regulation of Inflammation Response" pathway across different comparisons.

To elucidate the molecular mechanisms underlying the therapeutic effects of RBNPs and XDNPs in DSS-induced ulcerative colitis, we conducted a label-free quantitative (LFQ) proteomic analysis of colon tissue samples. The overall protein expression profiles across the Ctrl, Model, RBNPs, and XDNPs groups are depicted in the heatmap (Fig. 6A). The Model group displayed a markedly altered proteome relative to the healthy Ctrl group, reflecting extensive disruption of protein homeostasis during colitis. Treatment with RBNPs or XDNPs partially restored the proteomic landscape toward the Ctrl pattern, as confirmed by inter-sample correlation analysis (Fig. 6B) and PCA (Fig. 6C). The PCA plot revealed a clear separation between Ctrl and Model groups along PC1 (46.5% variance), with both treatment groups positioned closer to Ctrl, indicating mitigation of DSS-induced proteomic perturbations. Volcano plot showed 1570 differential expression proteins in the Model group relative to Ctrl (1083 upregulated and 487 downregulated) (Fig. 6D), highlighting the extensive molecular dysregulation in UC. XDNPs treatment reversed 1116 DEPs (248 upregulated and 868 downregulated), while RBNPs modulated 1476 DEPs (348 upregulated and 1128 downregulated) relative to the Model group (Fig. 6E and F), with many changes opposing those induced by DSS. Notably, RBNPs influenced a broader set of proteins, suggesting a potentially stronger or more comprehensive therapeutic effect. Treatment with XDNPs and RBNPs effectively reversed a significant proportion of the proteomic alterations induced by DSS, which correlates with the previously observed amelioration of colitis symptoms. Specifically, both nanoparticles concurrently upregulated 673 proteins that were likely suppressed by DSS challenge, underscoring a highly convergent mechanism of action between the natural and synthetic formulations (Fig. 6G). KEGG pathway analysis indicated that these restored proteins were predominantly enriched in metabolic pathways, providing a potential molecular basis for the recovery of body weight in treated mice (Fig. 6H). In contrast, proteins that were downregulated by both XDNPs and RBNPs, representing those aberrantly elevated in colitis, were significantly associated with immune and inflammatory processes. Key pathways modulated included platelet activation, leukocyte transendothelial migration, and NOD-like receptor signaling, reinforcing the premise that immunomodulation is central to their therapeutic efficacy (Fig. 6J). Further corroborating this, Gene Set Enrichment Analysis (GSEA) revealed a marked positive enrichment of the “Regulation of Inflammatory Response" pathway in DSS-treated mice, which was strikingly reversed by both XDNP and RBNP treatment, demonstrating their potent anti-inflammatory activity at the systems level (Fig. 6K). Collectively, these proteomic analyses demonstrate that XDNPs and RBNPs ameliorate DSS-induced colitis by reversing disease-specific proteomic alterations, primarily through suppression of inflammatory signaling. RBNPs exhibits a broader regulatory impact on the colonic proteome, suggesting a potentially more potent or extensive therapeutic mechanism.

### 3.7 Comprehensive evaluation confirms the *in vivo* safety profile of nanoparticles

**Fig. 7:**
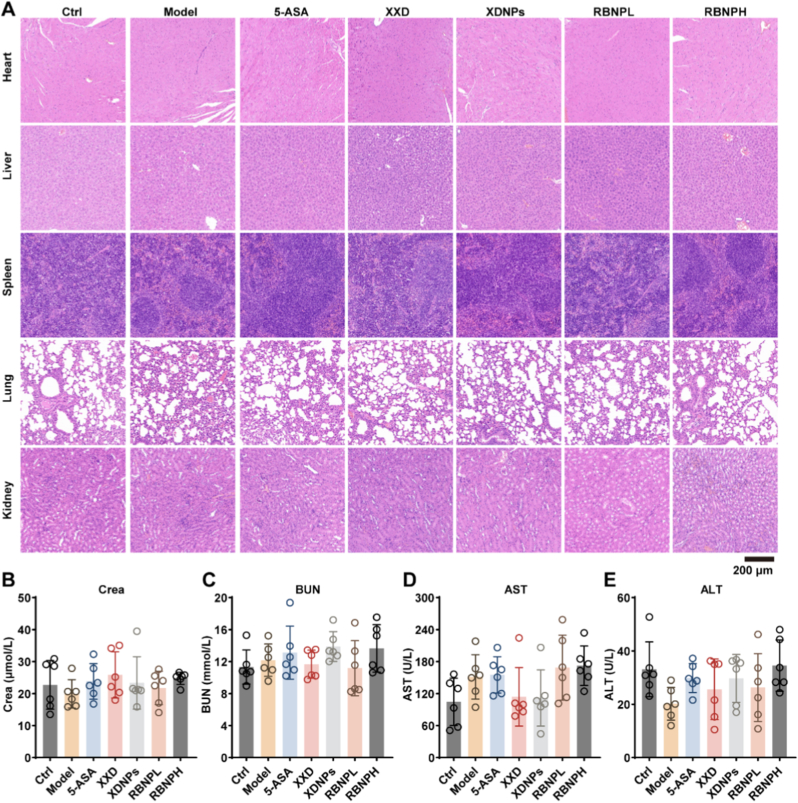
*In vivo* safety assessment of XXD, XDNPs, and RBNPs in mice. **(A)** Histopathological evaluation of major organs (heart, liver, spleen, lungs, and kidneys). Representative sections were stained with hematoxylin and eosin (H&E) to assess tissue morphology. **(B**–**E)** Serum biochemical analysis of key markers for liver and kidney function, including creatinine (B), blood urea nitrogen (C), aspartate aminotransferase (D), and alanine aminotransferase (E). Data are expressed as mean ± SD (*n* = 6). There was no significant difference between groups.

Prior to clinical translation, rigorous assessment of *in vivo* biocompatibility is essential. Building on the established safety of traditional XXD [[14], [15], [16]], we evaluated the systemic toxicity of XDNPs and RBNPs. Histopathological examination of major organs, including the heart, liver, spleen, lungs, and kidneys, revealed no treatment-related abnormalities. Hematoxylin and eosin (H&E) staining showed that tissue architecture and cellular morphology in mice treated with XXD, XDNPs, RBNPL, and RBNPH were indistinguishable from those of both Ctrl and Model groups, with no signs of necrosis, inflammation, degeneration, or abnormal cellular infiltration (Fig. 7A). To assess potential functional impairment of detoxification and excretory organs, key serum biomarkers were quantified. Renal function markers, creatinine (Crea) and blood urea nitrogen (BUN), and hepatic enzymes, alanine aminotransferase (ALT) and aspartate aminotransferase (AST), showed no statistically significant differences across Ctrl, Model, and treatment groups (Fig. 7B–E). All values remained within established normal ranges for healthy mice. Together, these complementary evaluations, including detailed histopathology and sensitive biochemical assays, demonstrate that both naturally derived XDNPs and synthetically assembled RBNPs exhibit excellent *in vivo* biocompatibility following oral administration. The absence of detectable adverse effects at the tested doses underscores the favorable safety profile of these nanoparticle formulations.

### 3.8 Nano-assembly enhances cellular uptake and anti-inflammatory efficacy *in vitro*

**Fig. 8:**
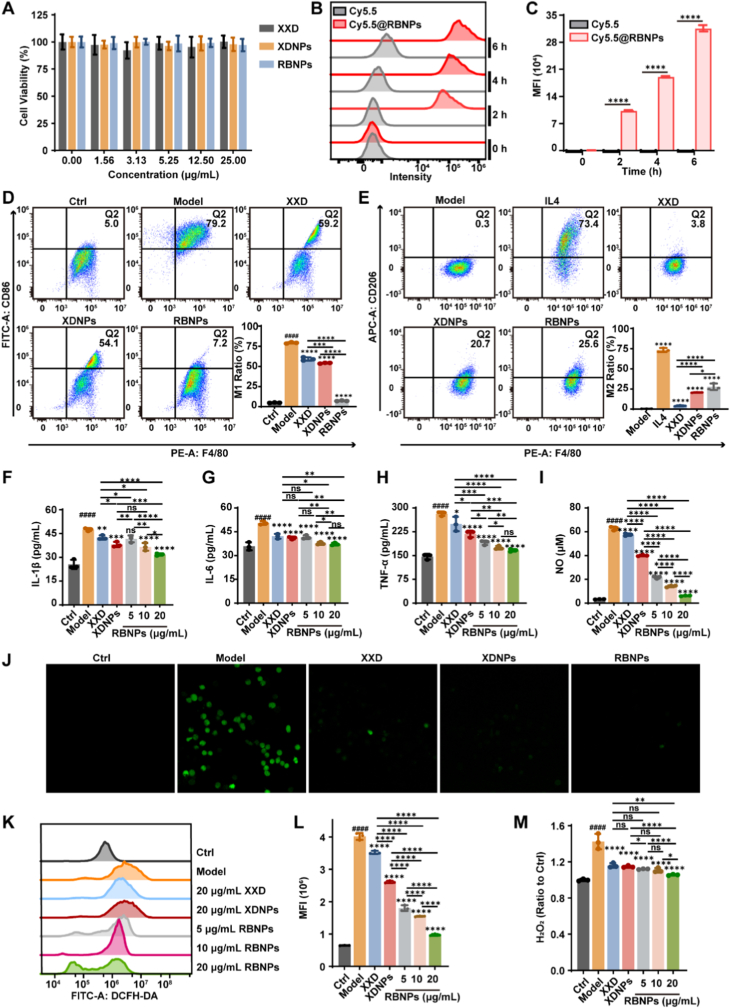
*In vitro anti-inflammatory effects of XXD-based self-assembled nanoparticles.***(A)** Viability of RAW264.7 cells after 24-h treatment with XXD, XDNPs, or RBNPs, assessed using the CCK-8 assay. **(B**–**C)** Flow cytometry analysis of cellular uptake of Cy5.5-labeled RBNPs (Cy5.5@RBNPs) versus free Cy5.5. **(D**–**E)** Flow cytometry assessment of macrophage polarization (M1/M2) in RAW264.7 cells following treatment with the indicated formulations. **(F**–**H)** ELISA quantification of pro-inflammatory cytokines IL-1β (F), IL-6 (G), and TNF-α (H) in culture supernatants. **(I)** Nitric oxide (NO) production in the supernatant. **(J)** Confocal laser scanning microscopy (CLSM) images. **(K**–**L)** Flow cytometry analysis of intracellular reactive oxygen species (ROS). **(M)** Intracellular hydrogen peroxide (H_2_O_2_) levels in LPS-stimulated RAW264.7 cells after treatment with the formulations. Data are presented as mean ± SD (*n* = 3). Statistical significance was calculated using one-way ANOVA with Tukey's post hoc test. ∗*p* < 0.05, ∗∗*p* < 0.01, ∗∗∗*p* < 0.001, ∗∗∗∗*p* < 0.0001 versus the Model group; ^####^*p* < 0.0001 versus the Ctrl group.

To validate the potent anti-inflammatory effects observed *in vivo* and elucidate the underlying cellular mechanisms, we conducted a series of experiments using RAW264.7 murine macrophages, a well-established model for inflammation research. We first evaluated the biocompatibility of the formulations with macrophages. Cytotoxicity assessment confirmed that XXD and the nanoparticles were safe at therapeutically relevant concentrations. RAW264.7 cells were treated with increasing concentrations (up to 25 μg/mL) of XXD, XDNPs, or RBNPs for 24 h. Cell viability, measured by the CCK-8 assay, remained above 90% across all concentrations tested compared to untreated controls (Fig. 8A), indicating excellent cytocompatibility within the intended dose range. A key advantage of nano-formulation is the potential for enhanced cellular internalization. To evaluate this, Cy5.5@RBNPs were prepared by incorporating a near-infrared fluorescent dye during nanoparticle synthesis. RAW264.7 cells were incubated with either Cy5.5@RBNPs or an equivalent concentration of free Cy5.5 for various durations. Flow cytometry analysis revealed a pronounced, time-dependent increase in cellular fluorescence in the Cy5.5@RBNPs group, indicative of efficient and progressive nanoparticle uptake (Fig. 8B and C). In contrast, cells treated with free Cy5.5 displayed minimal fluorescence at all time points, confirming that nano-encapsulation substantially enhances cellular delivery of the payload.

Based on the safety and uptake data, a concentration of 20 μg/mL was selected for subsequent functional assays. We first assessed macrophage polarization. Flow cytometry analysis (Fig. 8D and E) demonstrated that LPS stimulation strongly promoted M1 polarization (F4/80^+^CD86^+^). While XXD treatment partially mitigated this effect, XDNPs and RBNPs exhibited markedly stronger inhibition of M1 polarization (Fig. 8D). Concurrently, XXD treatment promoted M2 polarization (F4/80^+^CD206^+^) in macrophages, with XDNPs and RBNPs demonstrating more pronounced effects (Fig. 8E). These findings align with the superior immunomodulatory effects observed *in vivo*. Consistent with these findings, nanoparticle formulations more effectively suppressed the secretion of key pro-inflammatory cytokines. ELISA analysis of culture supernatants revealed that LPS robustly induced the release of IL-1β, IL-6, and TNF-α (Fig. 8F–H), whereas treatment with XDNPs and RBNPs significantly reduced the levels of all three cytokines compared to XXD at the same concentration. Reactive oxygen species (ROS) act as critical secondary messengers that amplify inflammatory signaling cascades [53,54]. We therefore examined the impact of the formulations on intracellular ROS generation (Fig. 8J–L) and the levels of nitric oxide (NO, Fig. 8I) and hydrogen peroxide (H_2_O_2_, Fig. 8M). LPS stimulation markedly increased intracellular ROS, NO, and H_2_O_2_ levels. All treatments, XXD, XDNPs, and RBNPs, significantly attenuated this oxidative burst. Notably, the nanoparticle formulations consistently demonstrated stronger inhibition of ROS and its derivatives compared to XXD, although statistical significance varied across assays. Collectively, these *in vitro* studies robustly corroborate the superior anti-inflammatory efficacy of XDNPs and RBNPs observed in the colitis model. The enhanced activity manifests across multiple dimensions: more effective suppression of M1 macrophage polarization and promotion of M2 macrophage polarization, greater inhibition of pro-inflammatory cytokine secretion, and stronger attenuation of ROS production. Importantly, the significantly improved cellular uptake of the nano-formulations provides a mechanistic explanation at the cellular level for their enhanced bioavailability and superior therapeutic performance relative to the native XXD decoction.

### 3.9 Identification and validation of VDAC1 as the direct target of berberine

**Fig. 9:**
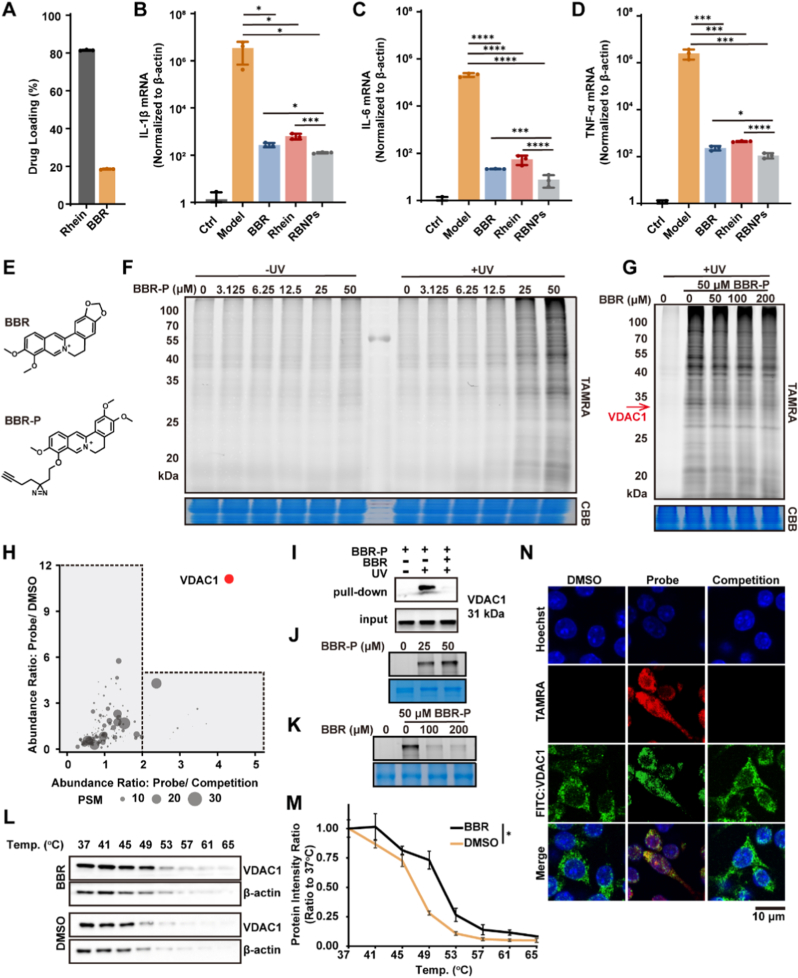
Identification and validation of VDAC1 as a molecular target of BBR in RAW264.7 cells. **(A)** Quantification of BBR and rhein loading in RBNPs by HPLC. **(B**–**D)** Relative mRNA expression of pro-inflammatory cytokines IL-1β (B), IL-6 (C), and TNF-α (D) in LPS-stimulated RAW264.7 cells. **(E)** Chemical structures of BBR and the alkyne-functionalized photoaffinity probe BBR-P. **(F)** In-gel fluorescence analysis of RAW264.7 cell lysates labeled *in situ* with BBR-P. **(G)** Competitive labeling demonstrating that excess free BBR reduces BBR-P protein labeling. **(H)** Scatter plot of the ratios of ABPP combined with TMT proteomics for quantitative identification of BBR-binding proteins. **(I)** Western blot after pull-down assay confirming the direct interaction between BBR-P and VDAC1. **(J)** In-gel fluorescence showing direct labeling of recombinant VDAC1 by BBR-P. **(K)** Competitive labeling demonstrating that excess BBR prevents BBR-P labeling of recombinant VDAC1. **(L**–**M)** Cellular thermal shift assay (CETSA) showing interactions between BBR and VDAC1: (L) representative Western blot and (M) melt curve quantification. **(N)** Confocal microscopy images illustrating co-localization of BBR-P with immune-stained VDAC1 in RAW264.7 cells. Data are presented as mean ± SD from three independent experiments. Statistical significance was assessed by one-way ANOVA with Tukey's post hoc test. ∗*p* < 0.05, ∗∗∗*p* < 0.001, ∗∗∗∗*p* < 0.0001 between the groups.

Following cellular internalization, self-assembled nanoparticles are expected to disassemble, releasing their constituent active molecules to engage intracellular targets [38]. To elucidate the molecular mechanism underlying the anti-inflammatory activity of RBNPs, we first sought to identify the principal bioactive component responsible for their efficacy. Comparative analysis revealed BBR as the dominant anti-inflammatory constituent within RBNPs. The anti-inflammatory potency of RBNPs (20 μg/mL), equivalent concentrations of free BBR (4 μg/mL), and free rhein (16 μg/mL) (based on drug loading determined by HPLC, Fig. 9A) was evaluated by measuring their effects on the mRNA expression of key pro-inflammatory cytokines (IL-1β, IL-6, TNF-α) in LPS-stimulated RAW264.7 macrophages. While both BBR and rhein exhibited inhibitory effects, the potency profile of BBR closely mirrored that of intact RBNPs and was significantly superior to rhein in suppressing all three cytokine transcripts (Fig. 9B–D). These findings establish BBR as the primary active component mediating the anti-inflammatory effects of RBNPs.

To identify the direct protein targets of BBR, we employed ABPP using a rationally designed photoaffinity probe. ABPP is a powerful chemoproteomic approach for mapping the interactome of bioactive small molecules in complex biological systems [55]. We synthesized a BBR-based photoaffinity probe (BBR-P) by incorporating a photoreactive diazirine group and a bioorthogonal alkyne handle onto the BBR scaffold, without compromising its core pharmacophore (Fig. 9E). The functionality of BBR-P was first validated in live cells. RAW264.7 macrophages were incubated with BBR-P (50 μM), followed by UV irradiation to activate the diazirine, inducing covalent crosslinking to proximal interacting proteins. Subsequent Cu(I)-catalyzed azide-alkyne cycloaddition (CuAAC) with TAMRA-PEG_3_-N_3_ enabled fluorescent visualization of labeled proteins via SDS-PAGE. Fluorescence scanning revealed distinct protein bands only in samples exposed to both BBR-P and UV light, confirming UV-dependent covalent capture of BBR-P targets (Fig. 9F). Pre-incubation of cells with an excess of native BBR prior to BBR-P addition and UV crosslinking substantially reduced fluorescent labeling intensity (Fig. 9G), demonstrating dose-dependent competition and confirming that BBR-P engages the same endogenous targets as the parent molecule.

For target identification, quantitative chemoproteomics was performed. BBR-P-labeled proteins in cell lysates were conjugated to biotin-azide via CuAAC, enriched with avidin agarose beads, digested with trypsin, and subjected to tandem mass tag (TMT) labeling followed by LC-MS/MS analysis. This approach identified 268 proteins enriched in the BBR-P (+UV) group compared to vehicle (-UV) controls. Applying stringent bioinformatic filters (FDR <0.05, fold-change >5 vs DMSO control; fold-change >2 vs BBR competition; peptide spectrum matching >2) yielded a high-confidence list of putative BBR targets. Among these, VDAC1 emerged as the top candidate, exhibiting the highest enrichment score and strong statistical significance (Fig. 9H). The direct interaction between BBR and VDAC1 was further validated using complementary orthogonal approaches. *In situ* competition in RAW264.7 cells and *in vitro* binding assays with recombinant VDAC1 consistently demonstrated that native BBR dose-dependently inhibited the binding of BBR-P to VDAC1 (Fig. 9I–K), confirming direct and specific engagement. To determine whether BBR selectively targets VDAC1 among the three VDAC isoforms, we performed pull-down assays followed by Western blot analysis specific for VDAC1, VDAC2, and VDAC3. The results confirmed that BBR binds preferentially to VDAC1, with minimal interaction detected for VDAC2 or VDAC3 (Fig. S6). CETSA showed that BBR treatment significantly increased the thermal denaturation temperature of VDAC1 compared to DMSO controls (Fig. 9L and M), providing direct evidence of BBR-VDAC1 binding within the cellular environment and stabilization of the protein structure. Confocal microscopy further confirmed that BBR-P co-localized with immunostained VDAC1 in RAW264.7 cells, and this co-localization was effectively competed away by excess BBR (Fig. 9N), reinforcing VDAC1 as a *bona fide* cellular target. Collectively, this multi-faceted validation strategy provides compelling evidence that VDAC1 is a primary and direct cellular target of BBR. Given that VDAC1 is a key mitochondrial outer membrane protein regulating metabolite transport and mitochondrial function, its identification provides a critical mechanistic link explaining the potent anti-inflammatory effects of BBR-containing nanoparticles.

### 3.10 BBR targets VDAC1 to inhibit mtDNA release and block NLRP3 inflammasome activation

**Fig. 10:**
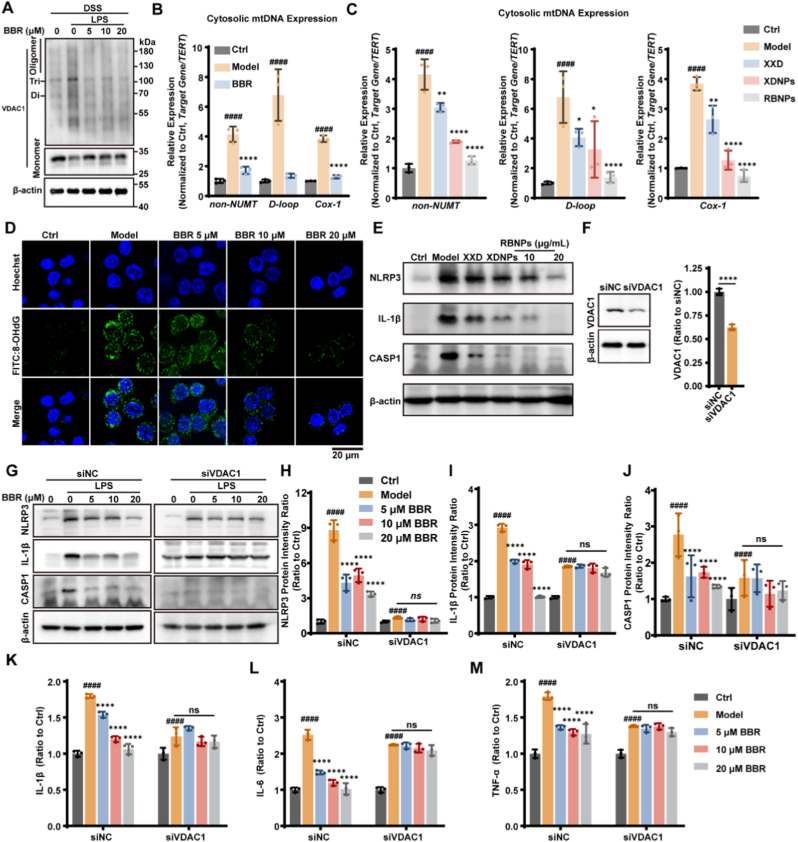
BBR inhibits cytosolic mtDNA release and NLRP3 inflammasome activation via VDAC1 in RAW264.7 cells. **(A)** Western blot analysis of VDAC1 oligomerization in LPS-stimulated RAW264.7 cells treated with increasing concentrations of BBR. **(B)** Quantification of cytosolic mtDNA levels in LPS-stimulated RAW264.7 cells following treatment with 20 μM BBR. **(C)** Quantification of cytosolic mtDNA levels in LPS-stimulated RAW264.7 cells treated with 20 μg/mL of XXD, XDNPs, or RBNPs. **(D)** Representative immunofluorescence images of RAW264.7 cells co-stained for 8-OHdG (oxidative DNA damage marker) and Hoechst nuclear stain, with or without LPS stimulation. **(E)** Western blot analysis of NLRP3 inflammasome activation markers in differentiated RAW264.7 cells treated with BBR. **(F)** Validation of VDAC1 knockdown efficiency by Western blot in RAW264.7 cells. **(G**–**J)** Western blot (G) and quantification of NLRP3 (H), cleaved IL-1β (I), and caspase-1 (CASP1, J) in control (siNC) and VDAC1-knockdown (siVDAC1) cells stimulated with LPS and treated with or without BBR. **(K**–**M)** ELISA measurement of IL-1β (K), IL-6 (L), and TNF-α (M) in supernatants from control and VDAC1-knockdown cells under the indicated treatments. Data are presented as mean ± SD from three independent experiments. Statistical significance was assessed by one-way ANOVA with Tukey's post hoc test. ∗*p* < 0.05, ∗∗*p* < 0.01, ∗∗∗*p* < 0.001, ∗∗∗∗*p* < 0.0001 versus Model group; ^####^*p* < 0.0001 versus Ctrl group; ns, not significant.

Having identified VDAC1 as the direct target of BBR, we next investigated the downstream molecular cascade responsible for its potent anti-inflammatory effects. Under cellular stress, mitochondrial VDAC1 oligomerization forms pores in the outer mitochondrial membrane (OMM), facilitating the release of mtDNA into the cytosol. Immunoblot analysis revealed a significant increase in VDAC1 oligomerization in LPS-primed RAW264.7 macrophages, which was dose-dependently inhibited by BBR (Fig. 10A). Cytosolic mtDNA acts as a damage-associated molecular pattern (DAMP), and its oxidation to ox-mtDNA (marked by 8-hydroxy-2′-deoxyguanosine, 8-OHdG) serves as a potent ligand for NLRP3 inflammasome activation, triggering caspase-1 cleavage and subsequent IL-1β maturation [40]. By binding VDAC1, BBR inhibits its oligomerization and the consequent mtDNA release. LPS stimulation markedly increased cytosolic mtDNA levels, as quantified by qPCR using mitochondrial gene-specific primers, whereas pre-treatment with BBR significantly blocked this mtDNA efflux (Fig. 10B). Consistently, XXD, XDNPs, and RBNPs also effectively suppressed cytosolic mtDNA accumulation (Fig. 10C), indicating that their anti-inflammatory effects converge on the VDAC1-mtDNA axis. BBR further attenuated the formation of oxidized mtDNA. Owing to its proximity to the electron transport chain and limited repair capacity, mtDNA is highly susceptible to oxidative damage. Immunofluorescence staining for 8-OHdG revealed a pronounced increase in ox-mtDNA in LPS-stimulated macrophages, which was substantially mitigated by BBR treatment (Fig. 10D). Given that ox-mtDNA serves as a critical trigger for NLRP3 inflammasome assembly [39], we next investigated the impact of XXD and its derived nanoparticles on this pathway. Western blot analysis confirmed that treatment with XXD, XDNPs, or RBNPs significantly suppressed NLRP3 inflammasome activation, as evidenced by the reduced cleavage of caspase-1 and maturation of IL-1β (Fig. 10E). Consistently, XDNPs and RBNPs exhibited comparable inhibitory effects on NLRP3 inflammasome activation in UC tissues (Fig. S7). Notably, RBNPs exhibited superior inhibitory activity compared to both XXD and XDNPs and effectively attenuated this pathway in a dose-dependent manner.

To establish a causal role for VDAC1 in mediating BBR's effect, we employed siRNA-mediated knockdown. Transfection of RAW264.7 cells with siRNA targeting VDAC1 (siVDAC1) significantly reduced VDAC1 protein expression compared to control siRNA (siNC) (Fig. 10F). Notably, in VDAC1-knockdown cells, BBR's ability to inhibit NLRP3 inflammasome activation (Fig. 10G–J), assessed by levels of NLRP3 (Fig. 10H), mature IL-1β (Fig. 10I) and cleaved caspase-1 (Fig. 10J), was markedly attenuated. This genetic rescue experiment confirms that VDAC1 is essential for BBR-mediated suppression of this inflammatory cascade. Furthermore, ELISA analysis demonstrated that BBR significantly reduced LPS-induced secretion of IL-1β (Fig. 10K), IL-6 (Fig. 10L), and TNF-α (Fig. 10M) in RAW264.7 cells, whereas these inhibitory effects were abolished upon VDAC1 knockdown, underscoring VDAC1 as a critical mediator of BBR's anti-inflammatory activity. In summary, these findings delineate a precise molecular mechanism: BBR directly binds VDAC1 on the mitochondrial outer membrane, inhibiting its stress-induced oligomerization. This blockade prevents mtDNA release into the cytosol and reduces the formation of ox-mtDNA, thereby limiting NLRP3 inflammasome activation, caspase-1 cleavage, and IL-1β maturation. The consistent inhibition of this pathway by XXD, XDNPs, and RBNPs highlights VDAC1-mediated suppression of NLRP3 as the central mechanism underlying the therapeutic efficacy of these nanoparticle formulations in ulcerative colitis.

## 4 Discussion

UC remains a significant therapeutic challenge due to the limited efficacy and potential adverse effects of current standard treatments, highlighting the urgent need for novel therapeutic strategies. Emerging evidence suggests that self-assembled nanoparticles act as crucial bioactive vehicles in traditional decoctions [[23], [24], [25], [26]]. Accordingly, we revisited the classical TCM formula XXD through the lens of nanomedicine and identified naturally occurring, self-assembled nanoparticles (XDNPs) within the decoction as the primary bioactive components responsible for its therapeutic effects against experimental colitis. Notably, XDNPs demonstrated markedly superior therapeutic efficacy compared to the crude XXD extract, as evidenced by attenuated weight loss, reduced DAI scores, and preserved colon length. Mechanistically, these nano-formulations exert potent anti-inflammatory effects by directly targeting VDAC1, thereby blocking the downstream ox-mtDNA/NLRP3 inflammasome axis. Collectively, this study not only deciphers the supramolecular basis of XXD's pharmacological action but also establishes a rationally designed, traditional medicine-inspired nanoplatform for safe and effective UC therapy.

Furthermore, we developed and preclinically validated a rationally designed, carrier-free nanoparticle (RBNP) composed of the primary bioactive constituents of XXD, BBR and rhein. RBNPs exhibited superior therapeutic outcomes relative to both natural nanoparticles (XDNPs) and the unprocessed aqueous decoction (XXD), establishing a clear efficacy hierarchy (RBNPs > XDNPs > XXD). The enhanced anti-inflammatory potency of these nano-formulations, evidenced by their improved ability to modulate macrophage polarization and suppress pro-inflammatory cytokine cascades, can be largely attributed to their optimized pharmacokinetic properties. This nano-strategic approach addresses a critical limitation of TCM pharmacology: the poor oral bioavailability of many potent phytochemicals [56]. Key bioactive molecules in XXD, including alkaloids [34,57] and benzenoids [21,41,58], are inherently lipophilic. Although hydroxyl and carboxyl groups on these compounds can form hydrogen bonds, these functional groups paradoxically reduce aqueous solubility by increasing intermolecular cohesion and crystal lattice energy. Nano-assembly offers a robust solution to this challenge. The formation of supramolecular nanoparticles substantially increases the surface area of active compounds, enhancing interactions with aqueous environments and improving solubility. Specifically, electrostatic attraction between the cationic nitrogen of BBR and the anionic hydroxyl group of rhein, reinforced by intermolecular π-π stacking, drives the spontaneous formation of structurally stable nanoparticles. This assembly translates into significantly enhanced oral bioavailability and systemic exposure. At the molecular level, the co-assembly of BBR and rhein enables a potential anti-inflammatory mechanism: BBR mitigates oxidative stress and suppresses NF-κB and NLRP3 signaling [[17], [18], [19]], while rhein ameliorates intestinal injury and inflammation, partially via activation of poly (ADP-ribose) polymerase-γ (PARP-γ) [20,22], and also inhibits NF-κB signaling. The combined effects produce a concerted and amplified anti-inflammatory response that exceeds the activity of either compound alone. This strategic nano-co-delivery ensures synchronized pharmacokinetics and target engagement, resulting in markedly improved therapeutic outcomes compared to the crude decoction or isolated compounds. The development of such advanced nano-formulations is therefore critical for modernizing TCM, aligning traditional remedies with contemporary requirements for efficacy, safety, stability, and patient compliance.

To dissect the molecular basis of the potent anti-colitis effects exerted by XXD and its derived XDNPs, we employed an ABPP approach. This chemoproteomic strategy enabled the identification of VDAC1 as a *bona fide* direct target of BBR, the principal bioactive constituent of XDNPs, within the colonic inflammatory milieu of UC. BBR, a natural isoquinoline alkaloid, is recognized for its pleiotropic pharmacological effects, including anti-inflammatory, anti-diabetic, and anti-neoplastic activities [18]. However, the precise molecular targets mediating its therapeutic efficacy, particularly in inflammatory diseases, have remained incompletely defined. Historically, the anti-inflammatory effects of BBR have been largely attributed to downstream modulation of signaling cascades, including the NF-κB and NLRP3 pathways [17,19]. Here, by employing rationally designed RBNPs as a tool to deconstruct the multicomponent complexity of native XDNPs, our work advances this understanding from correlative observations to direct target engagement. Specifically, we demonstrate that BBR, the primary bioactive component of XDNPs, directly binds to VDAC1, thereby blocking its stress-induced oligomerization and subsequent pore formation. This is of significant pathological relevance, as VDAC1 oligomerization forms large pores in the outer mitochondrial membrane that facilitate the cytosolic release of mtDNA [59,60]. In active UC, cytosolic mtDNA functions as a potent DAMP, driving immune hyperactivation and triggering NLRP3 inflammasome signaling. Given that VDAC1 expression is upregulated in active UC, our results firmly establish it as a rational therapeutic target [60]. By directly binding VDAC1 and preventing pathological oligomer formation, BBR redefines its mechanism of action from a broadly anti-inflammatory compound to a precise modulator of mitochondrial pore dynamics. This upstream molecular event directly links BBR to the maintenance of mitochondrial integrity and the suppression of a key inflammatory cascade, thereby explaining the potent anti-inflammatory effects observed with XXD, XDNPs, and RBNPs.

Despite these compelling findings, several limitations of the current study warrant consideration and inform future research directions. First, although the synthetic RBNPs, composed of the core bioactive components BBR and rhein, effectively recapitulated the primary therapeutic activity of XDNPs against UC, the pharmacological profile of natural nanoparticles is likely more complex. Minor constituents within XDNPs may contribute synergistically to efficacy or modulate off-target effects. Consequently, the therapeutic landscape of RBNPs may not fully recapitulate that of XDNPs, and the biological roles of ancillary components merit further investigation. Future systematic fractionation and reconstitution strategies are imperative to comprehensively map the contributions of these trace components to the overall nanotherapeutic efficacy. Second, while our 16 S rRNA sequencing provides valuable phenotypic observations of gut microbiota structural shifts, the lack of functional metabolic data, particularly SCFAs, remains a limitation. Therefore, further metabolomic profiling is warranted to complement the current findings and fully elucidate the underlying host-microbe metabolic crosstalk. Third, although VDAC1 is identified as a critical direct target of BBR, the multi-component nature of both RBNPs and XDNPs suggests that their comprehensive therapeutic effects likely arise from poly-pharmacology. Other protein targets, particularly those engaged by rhein or additional constituents, are likely involved. In keeping with TCM principles emphasizing multi-target synergism, future studies are needed to delineate a complete interactome and mechanistic map for these nano-formulations. Fourth, our target identification strategy focused on the active small molecule BBR and did not fully account for nano-specific properties of XDNPs and RBNPs. As nanomedicines, their activity may be influenced by enhanced biodistribution, tissue-targeting capabilities, or dynamic protein corona formation, which can alter their biological identity and interactions. Decoupling nano-carrier effects from cargo pharmacology represents an important avenue for further investigation. Last, recognizing the critical gap between preclinical murine models and clinical application, future studies will prioritize the validation of these findings in humanized systems. To this end, we aim to establish sophisticated human-relevant models, including co-cultures of patient-derived intestinal organoids with autologous immune cells. Integration of these platforms with organ-on-a-chip microfluidic technologies will further enable dynamic, high-fidelity assessment of the therapeutic efficacy, mucosal barrier permeability, and immunomodulatory safety of XDNPs and RBNPs within a human-relevant microenvironment. Such approaches will be instrumental in bridging the translational divide and advancing these nanotherapeutics toward clinical application.

## 5 Conclusion

In summary, this study deciphers the supramolecular basis of the classical formula XXD, demonstrating that its naturally assembled nanoparticles (XDNPs) ameliorate UC by precisely modulating a mitochondrial-centric signaling axis. We identify VDAC1 as the direct functional target of BBR, the principal bioactive component within these innate nanocarriers. This discovery establishes a mechanistic link between the traditional use of XXD and the precise molecular control of mitochondrial integrity and inflammatory activation. BBR binding prevents pathological VDAC1 oligomerization, thereby inhibiting the cytosolic release of mitochondrial DNA and the subsequent activation of the NLRP3 inflammasome in macrophages. The successful construction of carrier-free biomimetic RBNPs not only validated this mechanism but also underscored the critical role of the supramolecular nanostructure inherent to XDNPs. This unique architecture proved indispensable for overcoming the poor bioavailability of the crude extract, facilitating enhanced cellular uptake and potentiated mucosal immunomodulatory effects that collectively drive the superior therapeutic outcomes. Comprehensive *in vitro* and *in vivo* evaluations demonstrate excellent biocompatibility and systemic safety, highlighting the translational potential of these formulations. While further studies are required to explore minor constituents, additional protein targets, and the pharmacokinetics of XDNPs, our findings establish a mechanistic framework linking traditional medicine to modern nanotherapeutics. Collectively, this work not only elucidates a novel VDAC1-mediated anti-inflammatory pathway but also provides a rational design strategy for developing highly effective, biocompatible nanomedicines inspired by traditional herbal formulations, offering a promising platform for precision therapies in ulcerative colitis and related inflammatory disorders.

## CRediT authorship contribution statement

**Zheng Chu:** Investigation, Visualization, Writing – original draft. **Tong Yang:** Investigation, Visualization, Writing – original draft. **Ying Zhang:** Investigation, Visualization, Writing – original draft. **Qianru Zhu:** Formal analysis. **Dawei Wang:** Formal analysis. **Hechen Tang:** Investigation, Visualization. **Guoxin Zhang:** Formal analysis, Methodology. **Mengyao Jiang:** Formal analysis, Methodology. **Lirun Zhou:** Formal analysis, Visualization. **Liting Xu:** Formal analysis. **Tianyun Fan:** Formal analysis. **Junzhe Zhang:** Investigation, Methodology. **Ang Ma:** Investigation, Methodology. **Chengchao Xu:** Conceptualization, Supervision, Writing – review & editing. **Jigang Wang:** Conceptualization, Resources, Supervision, Writing – review & editing. **Huan Tang:** Conceptualization, Resources, Supervision, Writing – review & editing.

## Declaration of competing interest

The authors declare that they have no known competing financial interests or personal relationships that could have appeared to influence the work reported in this paper.

## Data Availability

Data will be made available on request.
