# Supplementary material for: Self-assembled nanoparticles from Xiexin Decoction attenuate ulcerative colitis by targeting VDAC1-Mediated NLRP3 inflammasome activation
Source: Mater Today Bio. 2026 Mar 27;38:103078. doi: 10.1016/j.mtbio.2026.103078 (PMC13089187; doi:10.1016/j.mtbio.2026.103078)
Supplement: Multimedia component 1 [file mmc1.docx]

**Supporting Materials**

**Self-Assembled Nanoparticles from Xiexin Decoction Attenuate Ulcerative Colitis by Targeting VDAC1-Mediated NLRP3 Inflammasome Activation**


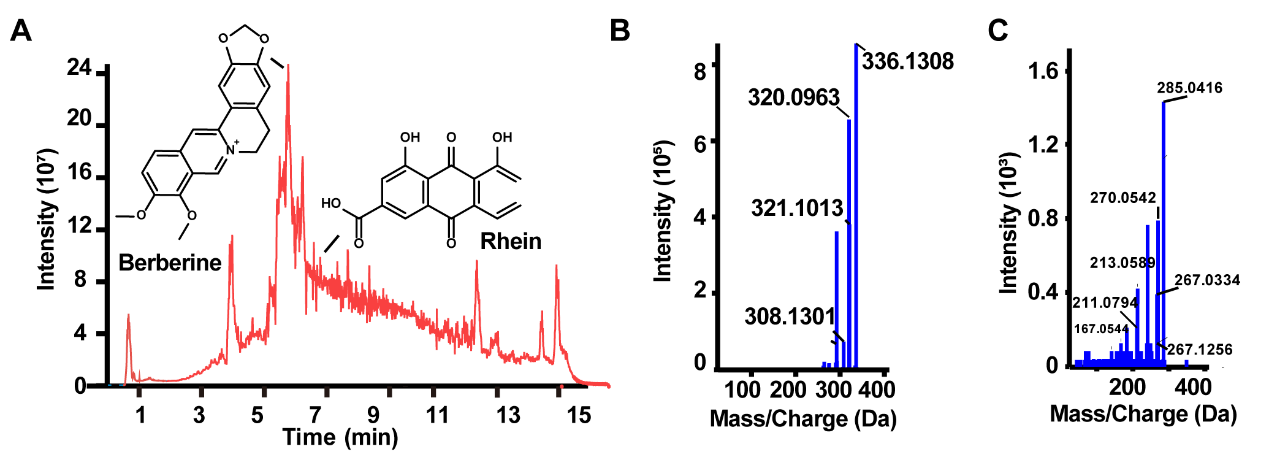


**Figure S1. UHPLC-QTOF-MS characterization of active components in XTNPs.**

(A) Total ion chromatogram of XTNPs acquired in positive ion mode. (B, C) Mass spectra of the identified components berberine (BBR) and rhein, respectively.


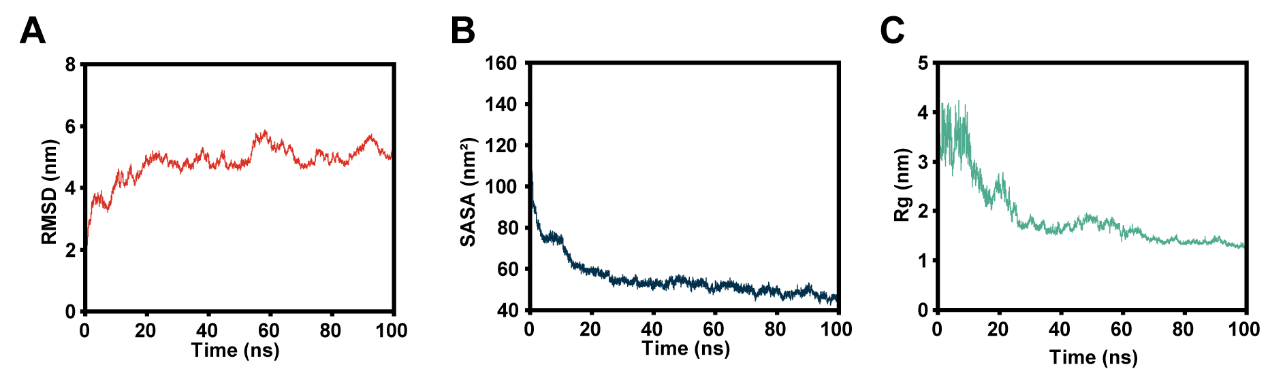


**Figure S2.** Root-mean-square deviation (RMSD) **(A)**, Solvent-accessibility surface area (SASA) **(B),** Radius of gyration (Rg) **(C)** of RBNPs changes over time under molecular simulation.


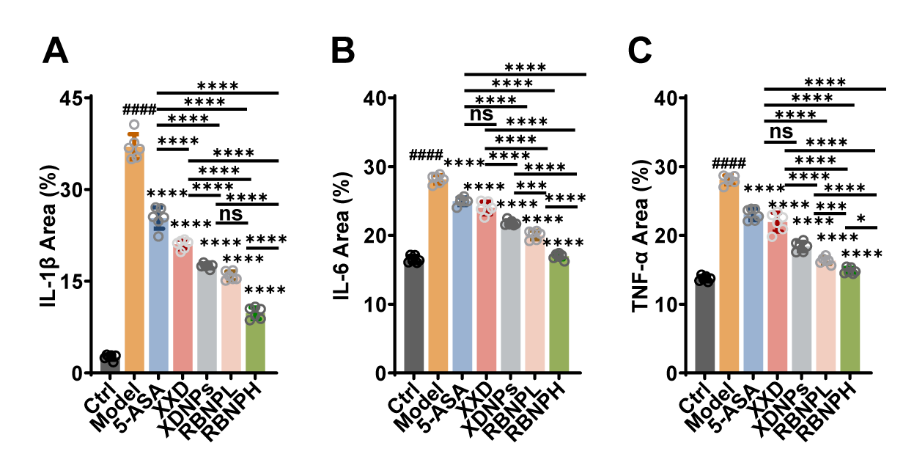


**Figure S3. IHC quantitative analysis of pro-inflammatory cytokine expression in the colon.**

The expression levels of IL-1β (A), IL-6 (B), and TNF-α (C) were quantified from IHC stained tissue sections (**Fig. 4G**). Data are presented as mean ± SD (*n* = 6). Statistical significance was assessed using one-way ANOVA with Tukey's post hoc test. **p* < 0.05, ***p* < 0.01, ****p* < 0.001, *****p* < 0.0001 compared to the model group, ####p<0.001 versus Ctrl group; ns, not significant.


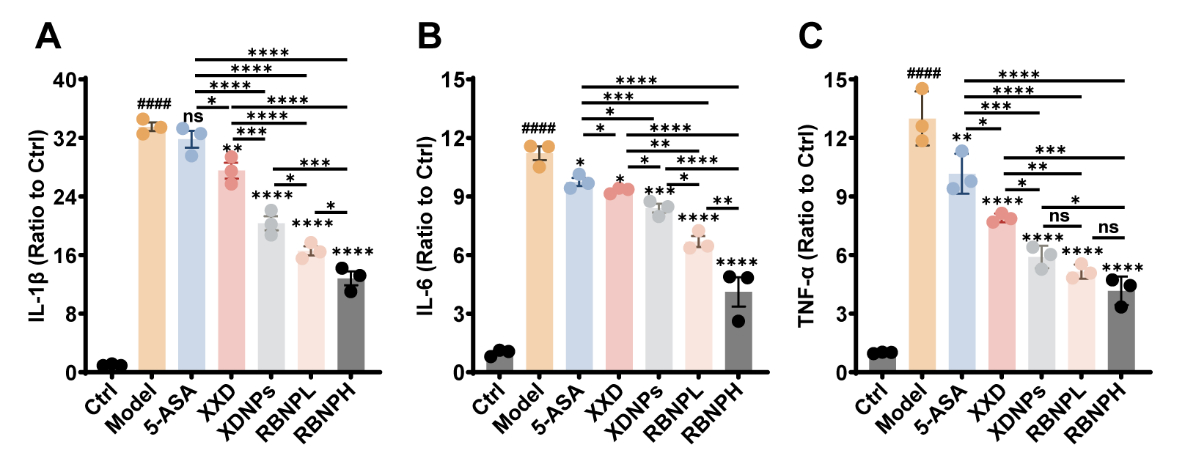


**Figure S4. Western blot quantitative analysis of pro-inflammatory cytokine expression in the colon.**

Western blot quantitative analysis of IL-1β (A), IL-6 (B), and TNF-α (C) in colon tissue lysates (*n* = 6 mice per group). Data represent the mean ± SD of three technical replicates from the samples. Statistical significance was determined using one-way ANOVA followed by Tukey’s post hoc test. *ns*, not significant, **p* < 0.05, ***p* < 0.01, ****p* < 0.001, *****p* < 0.0001 versus Model group; ####*p* < 0.001 versus Ctrl group.


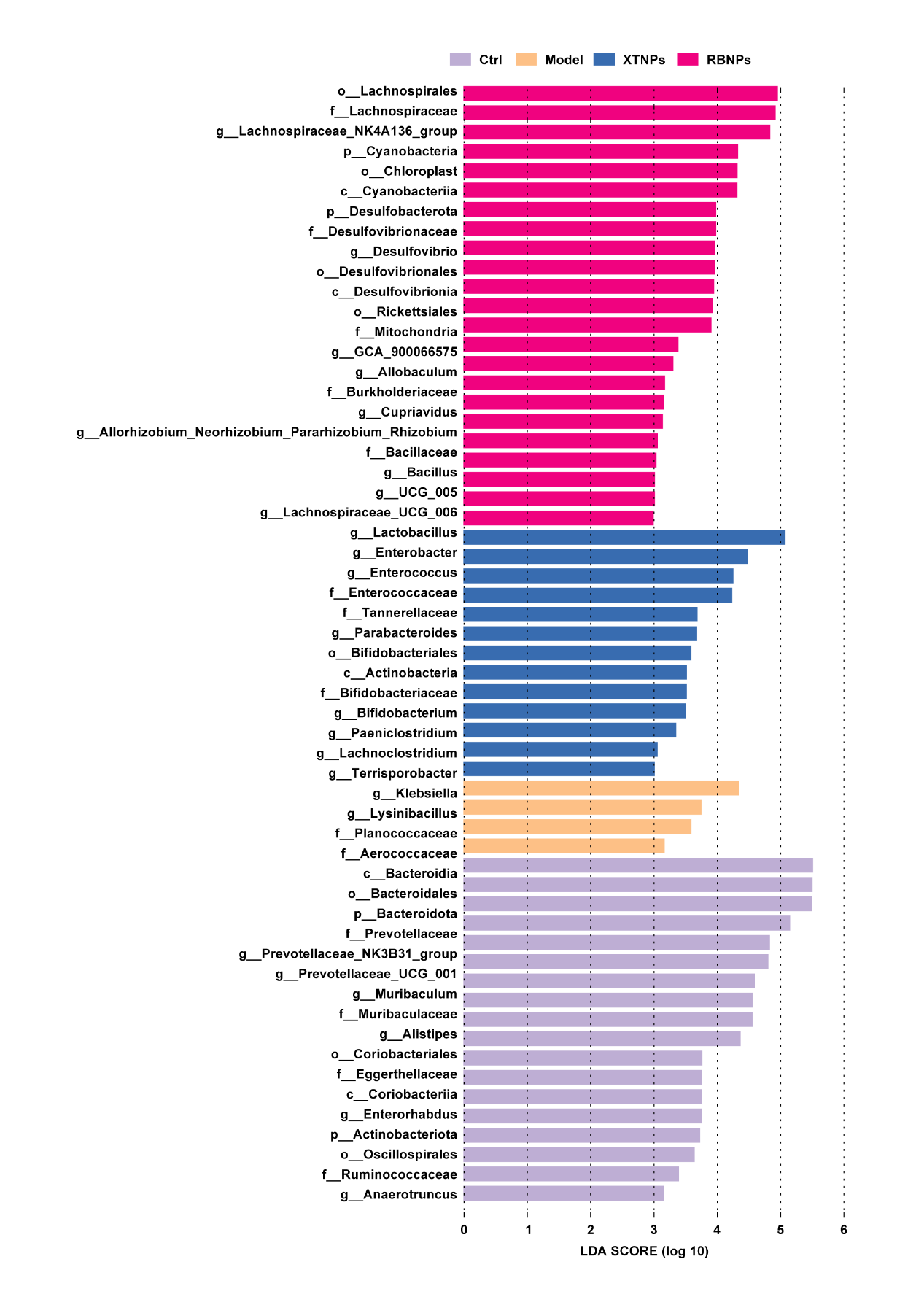


**Figure S5 Identification of differentially abundant microbial biomarkers using Linear Discriminant Analysis Effect Size (LEfSe).**


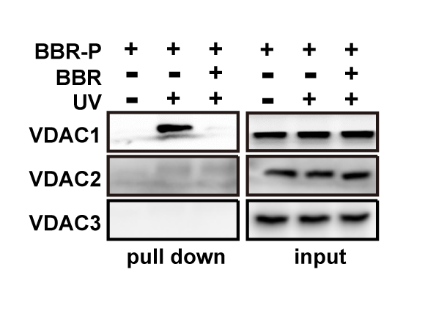


**Figure S6.** Pull-down assay for VDAC1, VDAC2, and VDAC3 in RAW264.7 cells using BBR-P.


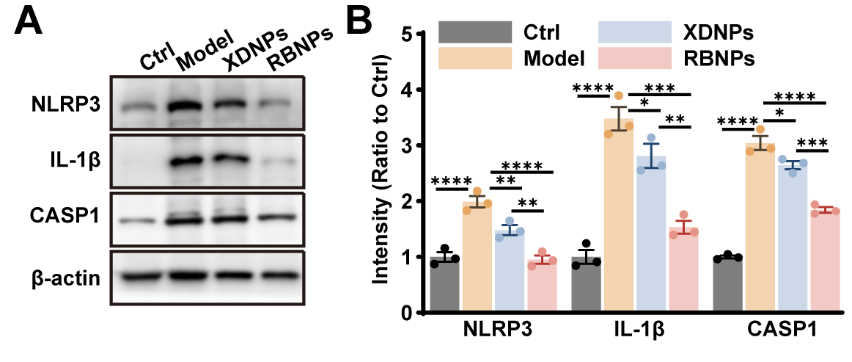


**Figure S7.** Western blot analysis of NLRP3 inflammasome activation markers in colon tissues from UC mice treated with XDNPs and RBNPs. **(A)** Western blot analysis of NLRP3, IL-1β, and CASP1 protein levels in pooled colon tissue lysates (*n* = 6 mice per group). **(B)** Relative quantification of protein band intensity (normalized to Ctrl). Data represent the mean ± SD of three technical replicates from the samples. Statistical significance was determined using one-way ANOVA followed by Tukey’s post hoc test. **p* < 0.05, ***p* < 0.01, ****p* < 0.001, *****p* < 0.0001 between the groups.

**Table S1 Sequence of primers for PCR**

| Gene | Sense (5'-3') | Anti-sense (3'-5') |
| --- | --- | --- |
| β-actin | CACCATGTACCCAGGCATTG | CCTGCTTGCTGATCCACATC |
| TNF-α | GCTGAGCTCAAACCCTGGTA | CGGACTCCGCAAAGTCTAAG |
| IL-1β | GCAACTGTTCCTGAACTCAACT | ATCTTTTGGGGTCCGTCAACT |
| IL-6 | TAGTCCTTCCTACCCCAATTTCC | TTGGTCCTTAGCCACTCCTTC |
| Tert | CTAGCT CATGTGTCAAGACCCTCTT | GCCAGCACGTTTCTCTCGTT |
| D-loop | AATCTACCATCCTCCGTGAAACC | TCAGTTTAGCTACCCCCAAGTTTAA |
| Cox-1 | GCCCCAGATATAGCATTCCC | GTTCATCCTGTTCCTGCTCC |
| Non-Numt | CCAGCTATCACCAAGCTCGT | CTAGAAACCCCGAAACCAAA |
